# Nuclear GSH import precedes coordinated cell cycle changes during regeneration

**DOI:** 10.1101/2023.11.28.569014

**Published:** 2023-12-13

**Authors:** Laura R Lee, Bruno Guillotin, Chanel Hutchison, Bénédicte Desvoyes, Crisanto Gutierrez, Kenneth David Birnbaum

**Affiliations:** New York University; New York University; University of Maryland; Centro de Biologia Molecular Severo Ochoa; Centro de Biologia Molecular Severo Ochoa

**Keywords:** Regeneration, Reactive Oxygen Species, Cell Cycle

## Abstract

Arabidopsis root tip regeneration requires cell division and cellular reprogramming. Here, we present new datasets that describe the cell cycle in Arabidopsis roots that maintain developmental context and cell-type resolution and provide an expanded set of cell cycle phase transcriptional markers. Using these data, we provide in vivo confirmation of a longstanding model in plants that glutathione (GSH) and reactive oxygen species (ROS) vary in a cell cycle dependent manner. We then demonstrate using long term time lapse imaging that cells in G1 phase undergo a transient peak of GSH prior to a tissue-wide coordinated entry into S phase. This coordinated S phase entry precedes a period of fast divisions, which we show appears to potentiate cellular reprogramming during regeneration. Taken together, this work demonstrates a role for GSH in coordinating cell cycle regulation and cellular reprogramming during regeneration.

## 1 Introduction

Plants have an incredible capacity to regenerate; entire organisms can regrow from a single somatic cell. The Arabidopsis root apical meristem (RAM) is an exemplary case of regeneration in plants because the organ can regenerate from fate-committed cells in the absence of stem cells or exogenous hormones [1]. This process requires the coordination of both cell divisions and cell identity changes. How these processes are coordinated remains an open question. Here, through transcriptional profiling of the cell cycle while maintaining developmental context, we confirm previous hypotheses that 1) individual cell types have unique cell cycle behaviors and 2) the expression of redox regulators changes throughout the cell cycle. We then demonstrate a role for antioxidants in coordinating regeneration and the cell cycle.

Cell cycle regulation has been studied extensively in Arabidopsis (reviewed in [2]). Many core cell cycle regulators are conserved between plants and animals (reviewed in [3,4]). The expansion of gene families in plants, such as the D type cyclins, allowed for developmental specialization. For instance, CYCLIN D-6 (CYCD6) is specifically expressed in some of the cells undergoing formative divisions such as cortical endodermal initial which gives rise to the cortical and endodermal cell files in the RAM [5]. CYCLIN D-7 (CYCD7) expression is restricted to the guard mother cell in the stomatal lineage and regulates a switch from asymmetric to symmetric divisions [6]. These examples suggest that cell type-specific regulation of the cell cycle in plants could be a more general trend, but one challenge facing the field has been studying the cell cycle in a way that maintains developmental context. Early transcriptional studies of the cell cycle in Arabidopsis employed synchronization of cultured cells [7,8], which provided valuable insight but could not provide cell type-specific information. In addition, cell synchronization techniques, such as sucrose starvation or double thymidine blocks, putatively align cells in G1 [7]. However, it is not clear if this aligned G1 is similar to G1 in vivo, or whether multiple G1 states exist, as has been proposed [9]. This may account for why the field currently lacks a reliable transcriptional marker for the G1 phase of the cell cycle, although CDT1a is a well-supported translational marker [10,11]. Therefore, questions remain regarding the relationship between cell cycle regulation and development in vivo.

Cell division is frequently coupled with cell identity specification. In the Arabidopsis sepal, giant cells are specified when MERISTEM LAYER 1 (ML1) expression exceeds a threshold level during G2/M phase of the cell cycle [12]. In the stomatal lineage, cell divisions are regulated by the expression of a series of master regulator basic helix-loop-helix (bHLH) transcription factors that concomitantly regulate cell identity (reviewed in [13]). In addition, cell cycle regulation is correlated with changes in cell fate specification, and this may be cell type-specific. In the root, a proximal-distal cell cycle duration gradient mirrors a differentiation gradient [14], and this is mainly due to differences in G1 phase length, wherein G1 duration becomes shorter the further shootward a cell is displaced from the quiescent center (QC) [15]. Alternatively, in the stomatal lineage G1 duration increases and cell cycles lengthen as cells commit to terminal differentiation [16]. Thus, cell types pair cell cycle regulation with fate specification in various ways in Arabidopsis. In the context of RAM regeneration, cell division is required [1]. However, cells can change identities without necessarily passing through a G1 phase [17]. As such, it is clear that cell fate is tightly linked to cell cycle regulation, but the mechanism through which these two processes are coordinated remains an open question.

**Fig. 1:**
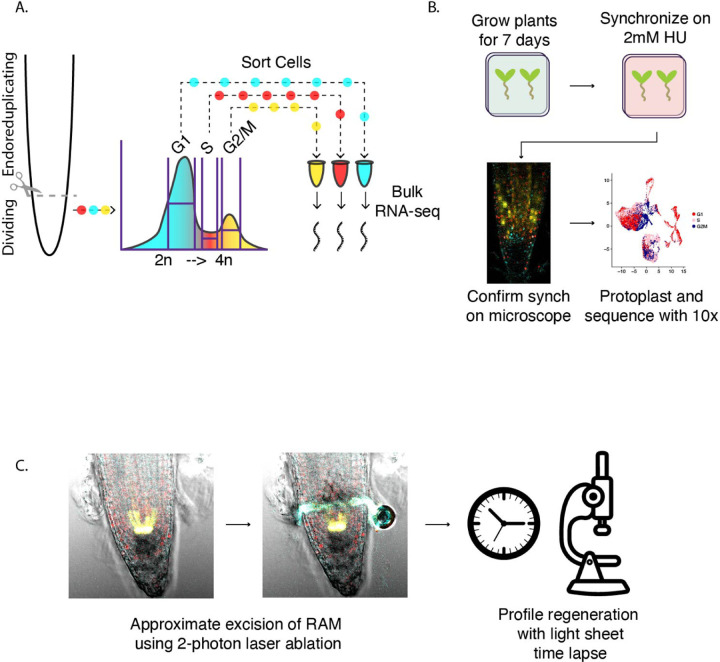
A. single cells colored coded by phase plotted in UMAP space. Libraries are separated by treatment condition. B. Differentially expressed genes ranked by the differential percentage of cells in each group expressing each gene. The highlighted genes are gold standard markers of phase-specific expression. The appropriate genes for each phase are highly ranked in the appropriate phase enriched library (x axis categories). C. Enriched GO terms for each top 50 marker set per phase grouped by semantic similarity. D. K means clustering of ploidy-sorted cells sequenced with bulk RNA-seq. Columns are the average expression of technical replicates, rows are genes. E. Intersected K means clusters with genes that are differentially expressed between phase-enriched single cell libraries, with the enriched library indicated by the color bar to the left of the heatmap. Broadly there is good agreement between the phase assignment of genes between these two methods. F. A dot plot showing the expression of gold standard cell cycle phase markers. Cells are grouped by phase assigned in Seurat using the top 50 genes most associated with S and G2M.

One potential signal that links cell cycle regulation and regeneration is redox stress. Injury results in accumulation of reactive oxygen species (ROS) that the plant must mediate in order to protect from further damage [18]. Glutathione (GSH) is the primary antioxidant in the cell [19]. GSH availability [20,21] and ROS patterning [22] in plants have previously been linked to root growth and cell cycle regulation [20,23,24]. GSH may be necessary for plant cells to pass the G1 to S transition [20] and nuclear ROS levels change cyclically in cell cycle-synchronized root tips [25]. GSH is enriched in the nucleus in division-competent cells in both plants and animals with the hypothesized function of protecting newly synthesized DNA from ROS-induced damage (reviewed in [26]). Bcl-2, the animal gene that imports GSH into the nucleus from the cytoplasm, is not conserved in Arabidopsis. However, evidence from Arabidopsis tissue culture does suggest that GSH is transported into the nucleus in a cell cycle-dependent manner with consequences for the redox state of both the nucleus and the cytoplasm [24]. Upon organ damage, plant cells also choose between committing to defense or regeneration by signaling through NPR1 [27], which is itself redox sensitive. Here we use two orthogonal methods to generate transcriptomic profiles of the cell cycle in the RAM while maintaining developmental context. In the first, we employ a hydroxyurea (HU) treatment time course to synchronize cells in the intact RAM [28] followed by single cell RNA-seq to generate phase-enriched profiles for the G1, S, and G2M phases of the cell cycle (Figure 1B). In the second, we perform fluorescence activated cell sorting (FACS) on cells from the mitotically active cells of the RAM using ploidy, which serves as a proxy for cell cycle phase (Figure 1A). Collective analysis of these datasets reveals 1) individual cell types have distinct cell cycle regulation at the transcriptional level and 2) the G1 phase of the cell cycle is uniquely tuned to respond to redox stress. In addition, we demonstrate disproportionate shortening of the G1 phase of the cell cycle occurs during RAM regeneration using long term time lapse microscopy (Figure 1C), and we correlate this shortening with new marker expression and tissue-wide ROS dynamics.

## 2 Results

**Fig. 2:**
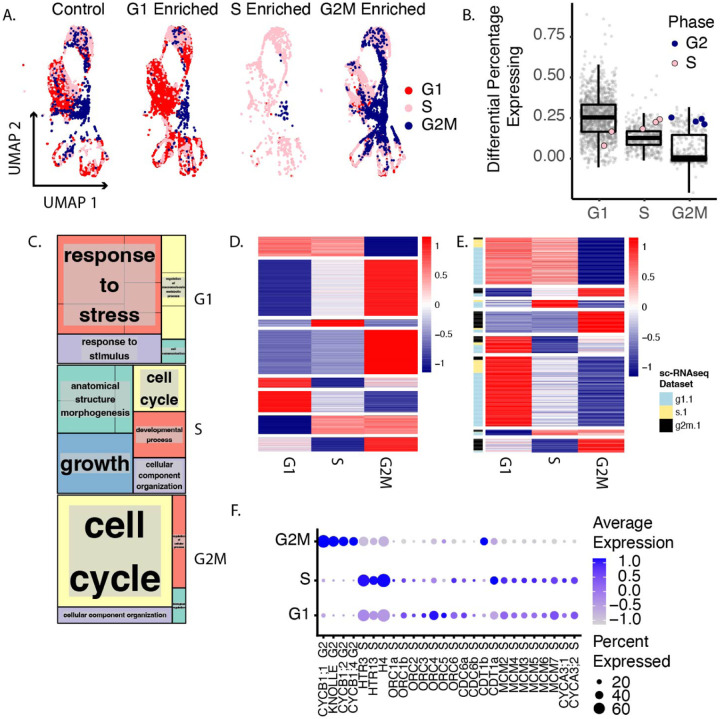
A. single cells colored coded by phase plotted in UMAP space. Libraries are separated by treatment condition. B. Differentially expressed genes ranked by the differential percentage of cells in each group expressing each gene. The highlighted genes are gold standard markers of phase-specific expression. The appropriate genes for each phase are highly ranked in the appropriate phase enriched library (x axis categories). C. Enriched GO terms for each top 50 marker set per phase grouped by semantic similarity. D. K means clustering of ploidy-sorted cells sequenced with bulk RNA-seq. Columns are the average expression of technical replicates, rows are genes. E. Intersected K means clusters with genes that are differentially expressed between phase-enriched single cell libraries, with the enriched library indicated by the color bar to the left of the heatmap. Broadly there is good agreement between the phase assignment of genes between these two methods. F. A dot plot showing the expression of gold standard cell cycle phase markers. Cells are grouped by phase assigned in Seurat using the top 50 genes most associated with S and G2M.

Transcriptional characterization of the cell cycle in Arabidopsis has primarily been done in tissue culture [7], or in synchronized Arabidopsis roots [28]. These techniques are powerful but they are limited in their ability to provide insight into in vivo cell cycle transcriptional information in a developmental/cell type-specific context. In particular, the G1 phase has been difficult to characterize because the existing markers for this phase are highly regulated post-translationally [10]. This phase is also likely to include various types of quiescent and dormant cells which may be functionally distinct from one another [9]. Synchronous plant cultures can be aligned in G1 by inducing states that do not occur in vivo such as sucrose starvation so this G1 may not be particularly transcriptionally similar to various kinds of G1 cells experience in plants. Here we used two orthogonal methods to characterize cell cycle transcriptomes in Arabidopsis while maintaining developmental context. In the first approach we applied the in vivo synchronization method [28] followed by single cell RNA-seq to obtain phase-enriched populations of cells where cell type-specific information is maintained (Figure 1B). We used these datasets to validate one another and to identify novel sets of phase-specific marker genes and then reanalyzed control single cell RNA-seq datasets using these marker genes. In a second, parallel approach, we used FACS to sort protoplasts by ploidy for bulk RNA-seq (Figure 1A). Cells were enriched for different phases of the cell cycle by synchronizing using HU treatment for varying durations (Figure 1B, 2A, Supplemental Figure 1). Phase enrichments were performed on the PlaCCI cell cycle reporter line [11] and confirmed with microscopy (Supplementary Figure 1). These conditions were then used to generate single cell RNA-seq libraries from which we defined marker genes for each phase de novo. We leveraged the power of single cell RNA-seq to account for any differential cell type representation between the phase-enriched libraries by downsampling each library so that all cell types were equally represented (Supplemental Figure 2). Differential expression was determined between each of these representation-normalized phase-enriched libraries. Statistically significant differentially expressed genes (p value ¡ 0.01) were then ranked by percentage of cells in that phase-enriched library expressing each gene (Figure 2B). This approach was validated by examining the ranking of “gold standard” cell cycle phase marker genes (Supplementary Table 1). We found that gold standard marker genes were more broadly expressed in the appropriate phase-enriched library (Figure 2B) where the y axis reflects the difference in the percentage of cells expressing a gene in a given scRNA-seq library versus all other libraries and the x axis reflects each phase-enriched library.

In parallel, and in order to control for any stress effects of HU treatment on the transcriptome, we also generated bulk RNA-seq libraries using cells sorted by ploidy as a proxy for cell cycle phase - cells in G1 have 2n ploidy while cells in G2M have 4n ploidy and cells with an intermediate amount of DNA are presumably in S phase [29]. This resulting dataset was analyzed by K-means clustering to reveal the expression patterns of highly variable genes among cell cycle phases (Figure 2D). We then explored the overlap in expression patterns between the bulk RNA-seq and the behavior of marker genes identified from the single cell RNA-seq (Figure 2E). We found that overwhelmingly these two approaches assigned genes to the same phase of the cell cycle. The top 50 genes per marker set derived from the single cell RNA-seq were chosen as phase markers in order to minimize the inclusion of genes that might behave in a cell type-specific manner. In addition, canonical cell cycle markers are lowly expressed (Supplemental Figure 3A) so they may not be the most informative genes for inferring cell cycle phase. The markers we selected are more broadly expressed (Supplemental Figure 3B). However, cyclins showed phase-specific gene expression once we assign cell cycle phases (Supplemental Figure 3C). GO enrichment analysis (Figure 2C) shows the functional enrichments of each phase marker set. As expected, cell cycle related terms are strongly represented in the G2M marker set. Similar term enrichments were found for the top 200 genes per marker set (Supplemental Figure 4), indicating this analysis is robust. Moving forward we use this top 50 set as our new phase marker set. We were surprised to find that terms relating to stress response were enriched in the G1 marker set. Among the terms contributing to this semantically similar cluster include “response to wounding” and “response to oxygen-containing compound”. Of note, G1 cells identified by ploidy are sorted from the same pool of protoplasts as the S and G2M cells, so there is no risk of batch effect in this experimental design. Therefore, we conclude that the stress terms we observe for the G1 markers are due to a biological trend rather than a technical artifact or batch effect.

Using our new phase marker set we assigned cells to phases in Seurat [30] and then looked at the expression of gold standard phase markers (Figure 2F). With these phase assignments, the origin recognition complex (ORC) family appears to be expressed more strongly in G1, while minichromosome maintenance complex (MCM) genes peak in S. This is consistent with the observation that ORCs are required in the pre-replication complex prior to MCMs (reviewed in [31]) and implies that this set of phase markers provides improved discrimination between G1 and S phase.

**Fig. 3:**
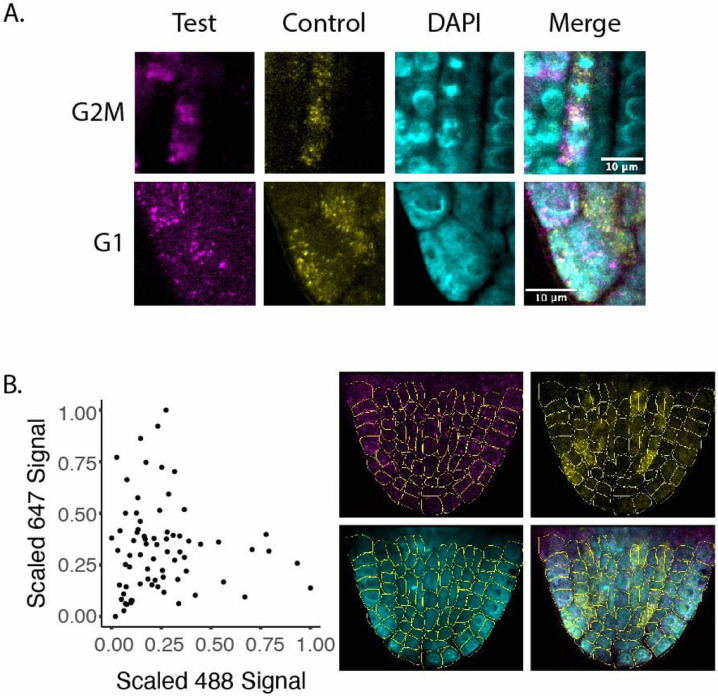
A. In situ hybridization for novel G1 and G2M probes. Known markers, shown in yellow, were hybridized with probes that fluoresce in the 488 nm range. Novel markers (magenta) were hybridized with probes that fluoresce in the 647 nm range. In the case of the G1 experiment, the control probe marks cells in S phase, as a strong G1 transcriptional marker was not already validated. B. Quantification of 488 and 647 signal for all cells in a recently emerged lateral root show an anticorrelation between cells with strong staining for the S phase probe (488) and the G1 phase probe (647). Segmented images are shown to the right.

In order to validate these markers in vivo we visualized transcripts directly using multicolor in situ hybridization (Figure 3A). This allowed us to simultaneously visualize a known phase marker in the same plant as a novel probe. Novel markers were selected for in situ experiments based on their high expression level and specificity, because such genes were most likely to be detected by in situ hybridization. We validated the novel G2M marker - AT4G23800 - by co-staining with a probe for CYCB1;1 and counterstaining with DAPI. Here we can see that both markers overlap in most cells. Additionally, both markers are present in cells with mitotic figures, as visualized by DAPI, further confirming the novel marker is expressed in cells in G2M phase.

To validate a novel G1 probe, we co-stained the new marker - AT5G21940 - with a well established S phase marker - AT5G10390 (HTR2). In this case we looked for both markers to be excluded from mitotic figures, as well as both markers to be anticorrelated with one another. The rationale for this approach is that the domains of G1 and S markers should be generally but not totally exclusive. We manually segmented all cells in a single optical plain in a recently emerged lateral root and then measured probe signal for each channel for each cell (Figure 3B). We then scaled the signal from 0 to 1 for all measurements within each channel because the two channels differed in brightness and found a strong anticorrelation between the scaled values. In addition, we classified 27 cells as having high staining for the G1 probe, 9 as high for the S probe, 5 as high for both, and 32 as high for neither. These results indicate the novel G1 probe we chose is present in the correct cells and therefore supports the conclusion that the G1 marker gene set we derived does reflect the G1 transcriptome.

**Fig. 4:**
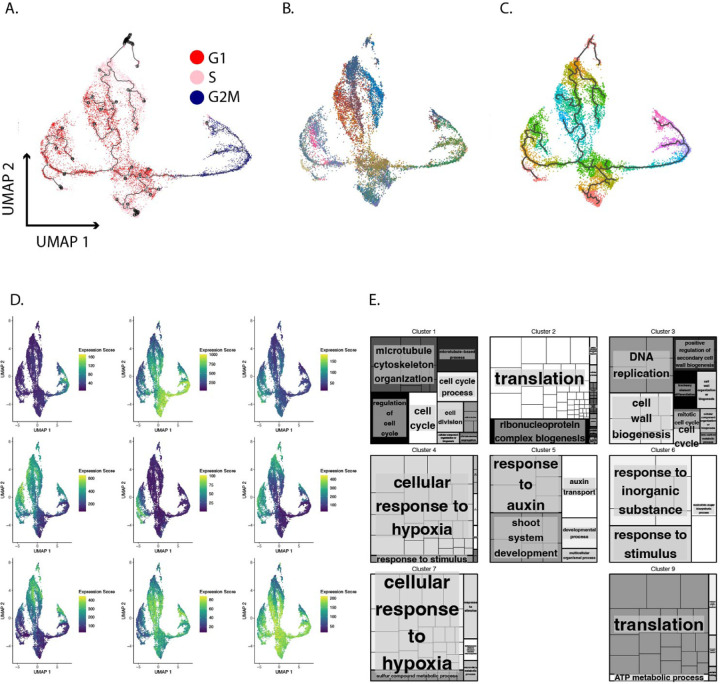
Cells were aligned in pseudotime using only genes highly associated with the cell cycle. A. UMAP of cells color coded by phase assigned in Seurat. B. UMAP of cells color coded by cell type. C. Cells are separated into 10 bins equally spaced along pseudotime. Cells are color coded to reflect their bin D. We performed hierarchical clustering of genes that are differentially expressed across psuedotime based on their expression in the bins shown in figure 4C. This resulted in identification of nine expression patterns shown here in UMAP space. Each UMAP shows the cumulative expression of a cluster of genes where blue is low and yellow is high. E. GO terms for genes in each expression cluster.

Some plant cell types commit to fates at different points in the cell cycle [12,16]. It has also been observed that different cell types in the RAM divide at different rates [14]. It is not known how these differences in cell division rate are mediated. One possibility is that different cell cycle behaviors are paired with different cell types in addition to different RAM zones [15]. We sought to test this hypothesis by aligning cells in pseudotime using Monocle3 [32–38]. To create the UMAP embedding we provided Monocle3 with only the top 150 genes most associated with the cell cycle. As expected, this produces an alignment in which cells are grouped by phase (Figure 4A). Interestingly, the resulting UMAP embedding indicated the existence of multiple paths through the cell cycle, with three distinct trajectories moving from G2 → M → G1 → S. The trajectory, anchored in G2M, proceeds to G1 where it splits into three separate trajectories that continue into S phase. In addition, we found that—despite filtering out cell type-specific markers—different cell types shown in different colors still inhabited distinct regions of the UMAP space (Figure 4B). In order to understand this transcriptional landscape more fully we used the graph test function of Monocole3 to identify genes with statistically significant, spatially distinct expression patterns within the pseudotime trajectory. We then divided the cells into 10 equally spaced bins based on pseudotime (Figure 4C), aggregated the expression of differentially expressed genes identified by the graph test within those bins, and then performed hierarchical clustering to reveal phase-specific gene expression patterns. The expression of genes in these clusters, visualized in UMAP space (Figure 4D), shows expression modules that are shared and distinct between phases, along with corresponding functional enrichments (Figure 4E). Thus, it appears that, while we could identify a universal set of phase markers, cells in different tissues showed variation in the expression of those markers. These patterns revealed signatures of cell type-specific cell cycles at the transcriptional level.

Gene expression that is distinct to G2M cells (cluster 1) are uniquely enriched for terms related to cell division. Gene expression that is shared between G2M and G1 cells (clusters 2 and 9) are enriched for terms related to translation, reflecting the growth functions of G1 and G2. Expression that is restricted to S phase cells (cluster 3) is indeed enriched for DNA replication terms. Expression that is strongly expressed in G1 and weakly expressed in S (clusters 4, 6, and 7) is enriched for terms related to hypoxia and oxidative stress. Gene expression specific to cells that appear will soon initiate endocycling (cluster 5) is enriched for auxin signaling and development terms. Interestingly, this cluster also includes the majority of the vasculature and ground tissue in the dataset (Figure 4B, 4E), indicating that these identities are adopted or are most clear transcriptionally when cells are preparing to become post-mitotic. There is considerable evidence in animals that events during the G1 phase of the cell cycle are critical for cell fate establishment [39–41], although it has been shown that, in at least some cases, cell identity changes in Arabidopsis in the context of injury repair do not strictly require passage through G1 [17]. Cell cycle speed is increased during RAM regeneration and our transcriptional results suggest that G1 phase is shortened relative to the other phases of the cell cycle during regeneration. Taken together, this raises the possibility that cells spend less time in G1 during regeneration in order to facilitate a cell fate change. While the scRNA-seq data allows us to infer the relative amounts of time cells spend in different phases based on the proportional representation of each phase in the dataset, it is not possible to infer the actual duration of the cell cycle or any given phase. Further, it is difficult to associate the duration of any given phase with a cell fate changing event. Therefore, to test this idea we first measured G1 phase duration concurrently with cell fate changes during regeneration using live imaging. Time lapse light sheet microscopy was chosen for its capacity to image over long time periods without causing bleaching or phototoxicity. We mounted seedlings in imaging cuvettes that were compatible with the Mizar light sheet microscope and used a 2 photon laser to generate transverse ablations that imitate RAM removal. We then imaged seedlings as a new RAM was established shootward of the ablation site over the next twelve or more hours, taking full z stacks in 4 colors every ten minutes. Seedlings successfully regenerate new RAMs under these conditions (Supplemental Figure 5). These experiments were performed on seedlings expressing the cell cycle marker PlaCCI [11] and the QC-columella marker WIP4 [42]. The time lapse allowed us to identify a regeneration zone immediately shootward of the ablation in which the new QC and other identities would be re-established based on the de novo expression of WIP4 over time. By monitoring this region, we could construct a time course over which reprogramming cells transitioned through the cell cycle.

**Fig. 5:**
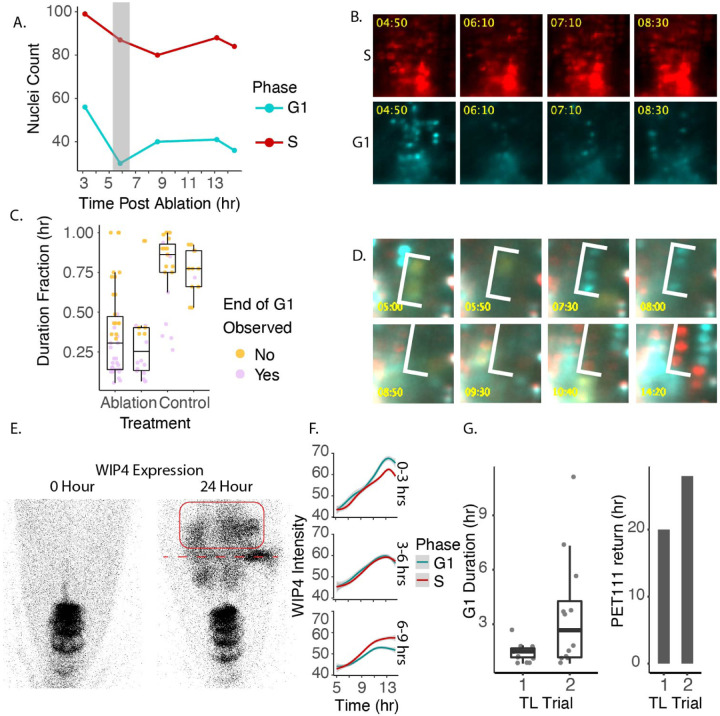
A. Quantification of the coordinated G1 exit, wherein cells leave G1 phase simultaneously around 6 HPA. B. Representative images of coordinated G1 exit. S phase cells, shown in red, serve here as a control to demonstrate that only the CFP signal disappears transiently during this time period. C. Quantification of G1 duration as a fraction of the total length of the time lapse. D. Representative example of a short G1 in which cells pass through the phase in as little as 2 hours. E. Representative image of WIP4 expression domain before ablation and also 24 HPA. The red box indicates the new WIP4 expression domain. The red dotted line marks the location of the ablation. F. Quantification of WIP4 signal over time in G1 and S phase cells. Cells are separated based on the observed G1 duration. G. Quantification of G1 duration alongside timing of PET111 expression establishment in the regeneration domain.

During the time lapse, we observed two key trends regarding G1. First we observed that cells in the regeneration zone coordinately exited G1 prior to new WIP4 expression (Figure 5A, 5B). Second, the number of G1 cells changes significantly over time (p-value = 0.008047) while the number of S cells does not (p-value = 0.3118; Chi Square test), suggesting that G1 duration is the most impacted during regeneration. This was followed by a period in which cells proceeded through G1 at an accelerated rate (Figure 5C, 5D). We measured G1 duration by marking the elapsed time between when CDT1a became visible after mitosis and when CDT1a was degraded, indicating S phase entry. The number and duration of G1 events is summarized in Table 1. Some of the observed G1 events did not end during the course of the time lapse in both the control (76 percent) and the ablation (38 percent) time lapse movies. In these cases we measured G1 duration in three ways: 1) as the time between when CDT1a became visible and the final frame of the time lapse, 2) as equal to the observed G1 duration time for this region of the root, which is estimated to be longer than 20 hours [15], and 3) as the fraction of total movie duration. By any of these metrics, the difference in G1 duration is statistically significant (p-value = 1.614e-08, p-value = 2.04e-05, or p-value = 3.221e-09, respectively, using the Mann-Whitney test). The specific, highly localized set of cells that will reprogram to generate the new root tip undergo much more rapid G1 than their neighbors. Together with previous results that showed a rapid overall cell cycle for regenerating cells, it appears that cells largely abbreviate G1 to speed the cell cycle.

Short G1 phases are a known feature of totipotent animal stem cells (reviewed in [43]).Therefore, we hypothesized that cells with short G1 phases would more efficiently reprogram during RAM regeneration. To determine if cells with short G1s showed any increased tendency to reprogram, we measured WIP4 intensity over time for all cells in the regeneration zone (Figure 5E, 5F). We categorized events based on their duration into short, medium, and long (Figure 5F). We found that while S phase cells gained WIP4 expression at a similar rate, regardless of S phase duration, cells with short G1 gained higher WIP4 expression levels than cells with long G1 (Figure 5F). We ruled out the possibility that this was due to noise in the data by performing the same analysis on a no-ablation control time lapse in the stable WIP4 domain, and found that there was no relationship between WIP4 expression and G1 duration (Supplemental Figure 6).

WIP4 is a marker that is re-established early during regeneration. It was possible that only early regeneration events were linked to G1 duration. To test this, we performed long term time lapses on regenerating roots, but this time we used a line in which PlaCCI was crossed to the mature columella marker, PET111. In these time lapses we measured G1 duration and the time to PET111 expression return (Figure 5G). We exploited the variability in PET111 return time and G1 duration between roots to explore whether these two variables were correlated. While not directly proportional, G1 duration was predictive of PET111 return time. A root in which the median G1 duration was 1.5 hours began to express PET111 in the regeneration domain at 20 HPA. A second root in which median G1 duration was 2.7 hours began to express PET111 at 28 HPA. We conclude that short G1 phases are strongly associated with reprogramming during regeneration.

While these data suggest G1 duration is linked to reprogramming efficiency, they do not establish a causal link. To establish a mechanistic relationship, we began by examining the functions of genes that were upregulated in G1. GO terms relating to stress were enriched for G1 phase gene expression in both the single cell and bulk ploidy sort datasets. Upon closer examination, it became clear that these terms specifically related to hypoxia and reactive oxygen species (ROS). GSH is the primary antioxidant in the cell, and we observed that many Glutathione S-transferases (GSTs) were preferentially expressed in the G1 phase as well. The cell cycle has been proposed to be a ROS cycle in both plants and animals [23], and ROS generation is a hallmark of tissue damage [18]. In addition, GSH has been demonstrated to be necessary for the G1→S transition in Arabidopsis [20]. Therefore, ROS dynamics may serve in part to coordinate the cell cycle and regeneration.

**Fig. 6:**
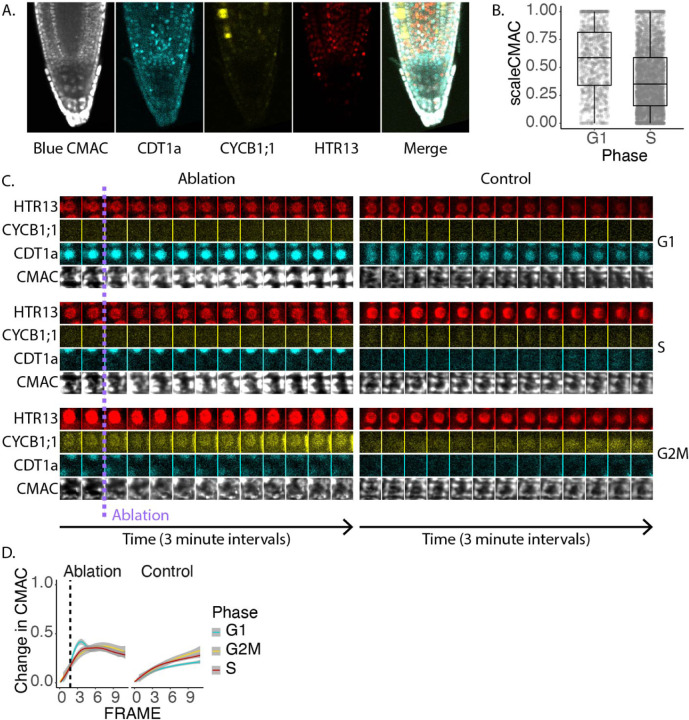
A. Representative confocal microscopy image of a PlaCCI seedling stained with blue CMAC overnight. B. Quantification of blue CMAC in G1 and S phase nuclei. Overall, G1 nuclei have higher blue CMAC levels than S phase nuclei. C. Time lapse data of representative cells from each phase of the cell cycle displayed as a montage. On the left are cells before and after a transverse ablation. On the right are control cells. D. Nuclear CMAC intensity following a transverse ablation or without ablation. Cells in different phases are plotted separately and CMAC intensity is represented as the change over the original value. Frames are three minutes apart. D. Quantification of the change in blue CMAC levels in nuclei of cells in each cell cycle phase in a control or an ablation time lapse. G1 cells in the ablation time lapse undergo a specific temporary increase in blue CMAC stain.

To explore this we decided to observe ROS dynamics in vivo in two ways. First, we performed live imaging with the ROS indicator, H2DCFDA, and using the ratiometric ROS reporter. Second, we monitored GSH levels during live imaging with the GSH stains, blue CMAC and CMFDA (Supplemental Figure 7). Each of these dyes are non-toxic and membrane permeable. In addition we also explored the consequences of GSH depletion on regeneration using the GSH synthesis-inhibitor, L -Buthioninesulfoximine (BSO), which has been validated to be effective in Arabidopsis [21]. These experiments were performed when possible using PlaCCI seedlings in order to gather cell cycle information simultaneously with ROS or GSH information. While blue CMAC has been available for some time, to our knowledge it has not been imaged with cellular resolution in the Arabidopsis RAM. It is immediately apparent that blue CMAC has cell file-specific staining patterns in the RAM. While the cap, epidermis, cortex, and endodermis stain brightly with blue CMAC, the stele stains much more faintly (Figure 6A). We do not believe this to be an artifact of dye penetration for two reasons. First, blue CMAC and CMFDA have similar staining patterns, while H2DCFDA stains all files relatively evenly (Supplemental Figure 7). However, the chemical structures of CMFDA and H2DCFDA are nearly identical, which suggests that they should have similar ability to penetrate the root [44]. With both blue CMAC and CMFDA we observe highly concentrated staining in the endodermis and cortex (Supplemental Figure 7A), and GSH biosynthesis genes are also highly expressed in these cell types (Supplemental Figure 7B). Therefore, we conclude that the outer files have a significantly higher GSH content than the stele.

In addition to imaging blue CMAC under normal conditions and following RAM excision, we also used time lapse confocal imaging paired with the same ablation strategy described above to observe changing GSH localization within the first 30 minutes of tissue damage. We found that in control roots, blue CMAC signal was higher in G1 phase nuclei than in S phase nuclei (Figure 6B). We further observed a pulse of nuclear GSH immediately after ablation that was most prevalent in G1 phase cells (Figure 6C, 6D). This coincides with the fact that nuclear GSH is high immediately following manual RAM excision but decreases by 9 HPC (Figure 6D), and at each of these timepoints G1 cells maintain higher GSH signal than S phase cells overall (Figure 6E). Intriguingly, in the 2 and 4 HPC time points, nuclei did maintain higher normalized CMAC signal shootward of the excision site in a pattern that correlates with the region in which cells undergo fast G1 phases (Supplemental Figure 8), suggesting that GSH is required in nuclei early during regeneration.

**Fig. 7:**
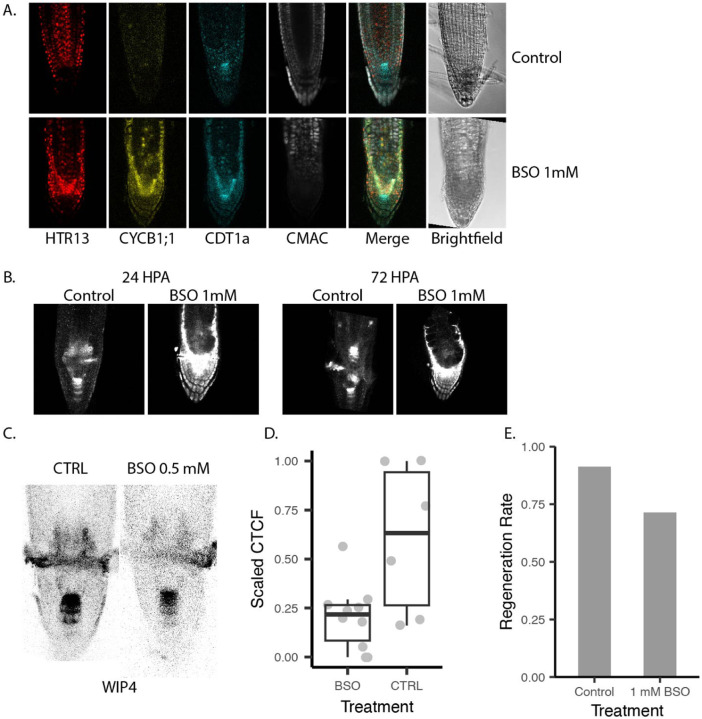
A. 7 DPG seedlings (PlaCCI × WIP4) grown on normal 1/2MS (ctrl) or germinated on 1/2MS+1mM BSO then stained overnight with blue CMAC. B. WIP4 signal in a median section of a control and a BSO-treated root at 24 and 72 HPA. C. Representative images of CMAC and WIP4 signal 24 HPA. D. Quantification WIP4 intensities in the regeneration zone 24 HPA. E. Gravitropism rates of roots grown on standard ½ MS or transferred to ½ MS with 1 mM BSO for 48 hours prior to meristem removal.

We next asked whether perturbing the ability of roots to buffer ROS could inhibit regeneration. We treated seedlings with the GSH synthesis inhibitor L-Buthionine-(S,R)-sulfoximine (BSO) by either 1) transferring seedlings to media containing 1mM BSO at 7 DPG or 2) germinating seedlings on plates supplemented with 1mM BSO, or 3) germinating seedlings on media supplemented with 0.5 mM BSO. Germinating seedlings on media containing 1mM BSO resulted in strongly perturbed root morphology as previously reported [21] and a depletion of blue CMAC staining signal (Figure 7A). We performed this experiment on seedlings expressing PlaCCI and a WIP4 transcriptional reporter in order to simultaneously track cell division and QC reestablishment. We observed that the majority of seedlings treated in this manner had short roots and low CMAC signal. We performed ablations on control seedlings and BSO-treated seedlings and observed them over a regeneration time course via confocal microscopy for 72 hours. The control seedlings regenerated a new QC shootward of the ablation over the course of 72 hours (Figure 7B). Despite dramatic morphological changes, cells in BSO-treated roots showed some weak WIP4 expression shootward of the ablation at 24 hrs HPA that remained constant until 72 HPA and failed to form an expression pattern indicative of new QC establishment (Figure 7B).

Roots germinated on a lower concentration of BSO had overall normal morphology as previously reported [21]. While BSO-treated roots did eventually regenerate, they had a lower amount of WIP4 expression in the regeneration zone 24 HPA (Figure 7C, 7D). Roots transferred to 1 mM BSO for two days prior to RAM excision also showed a slight depletion in regeneration rate as measured by gravitropism (Figure 7E). Thus, perturbing GSH levels impaired regeneration and cellular programming, consistent with a model in which GSH’s role in promoting G1 exit is a critical step in regeneration.

## 3 Discussion

There is a profound connection between cell division and cell fate specification across the kingdoms of life. This link in the context of root tip regeneration is particularly fascinating, given that fate-specified cells in roots must reprogram in order for regeneration to occur. Here, we leveraged the ability to induce cellular reprogramming, alongside the ability to closely monitor cells with both time lapse microscopy and transcriptomics, to demonstrate that the rate of cell division has a direct consequence on cellular reprogramming.

Using bulk and single cell RNA-seq we defined a list of cell cycle phase markers with in vivo relevance, including a large set of G1 markers, which were previously unavailable in the Arabidopsis root. This data established in vivo support for the hypothesis that plant cells differentially express ROS response genes across the cell cycle, indicating the cell cycle is linked to ROS homeostasis in roots. In parallel, we demonstrated that coordinated G1 exit and fast cell cycles occur during regeneration in vivo. We also correlate G1 speed with regeneration speed, which suggests fast cell divisions potentiate cellular reprogramming during regeneration. Unifying these observations, we show with microscopy that G1 nuclei have higher GSH levels and import GSH more quickly after tissue damage than cells in other phases. Longstanding evidence suggests that nuclear GSH import is required for licensing the G1 to S transition [45]. Taken together, this suggests a model in which a spike in nuclear GSH that occurs after tissue damage is responsible for the coordinated G1 exit we observe early in regeneration, after which fast divisions begin. The mechanistic basis of the link between G1 duration and regeneration via antioxidant regulation that we establish here suggests future research directions. Each tissue in the plant appears to maintain a unique relationship between cell division timing and cell fate specification. Therefore, it would be interesting to know whether this relationship is maintained in other developmental contexts in which tissue damage may occur. While we show that antioxidant availability is necessary to allow for regeneration to occur in Arabidopsis, it would be fascinating to know whether this relationship is consistent in other plant species, particularly crops.

There are circumstances in both plant and animal systems where GSH is imported into the nucleus. In animals, GSH is imported into the nucleus through Bcl2-associated athanogene pores [46], but the Bcl-2 gene is not conserved in plants. We demonstrate in this work that GSH is enriched in G1 nuclei in vivo and that G1 nuclei more quickly import free GSH than nuclei in cells in other phases of the cell cycle. We do not know what nuclear membrane property in plants allows for this to happen. One reasonable hypothesis is that G1 nuclei feature a differential permeability of the nuclear envelope. However, an exciting hypothesis is that plants evolved an independent transporter mechanism to bring GSH into the nucleus. Such a case of convergent evolution would indicate that a ROS cycle within the cell cycle, as was earlier proposed [23], is critical across the kingdoms of life.

Our work suggests there is a relationship between the burst of GSH in the nucleus that occurs immediately after tissue damage and the coordinated G1 exit observed approximately 6 hours later. However, it is not clear what mechanism exists that could directly link these two temporally disparate events. Given that the conservation of nuclear/cytoplasmic GSH cycling throughout the cell cycle is conserved between species, it seems likely that there is a mechanism that senses either GSH or ROS within nuclei directly. Such a mechanism is not well understood in any organism [47]. Given the relationship between nuclear ROS, cell cycle regulation, and cell identity changes, delineation of such a mechanism is critical for developmental biology broadly.

## 4 Materials and Methods

### 4.1 Plant growth and treatment conditions

Seedlings were grown vertically in an incubator set to long day conditions on ½ MS media unless otherwise noted. For HU treatment seedlings were synchronized in one of three cell cycle phases as previously described [28]. Briefly, seedlings were grown until 6 DPG vertically on 1/2 MS on top of sterile mesh. Then seedlings were transferred to 1/2 MS plates supplemented with 2mM HU. Various incubation times were used to synchronize cells in different phases of the cell cycle as follows: 6 hours for S phase, 17 hours for G2M, and 22 hours for G1. Synchronization in each phase was confirmed via confocal microscopy using the PlaCCI reporter. For BSO treatment seedlings were germinated on 1/2 MS media alone (control) or supplemented with 1 or 0.25 mM BSO. as previously described [21]. They were grown vertically on this media until they were 7 DPG and then used for either imaging or regeneration assays. PlaCCI seedlings were crossed to cell type reporters including WIP4 (columella and QC) and WOX5 (QC), PET111 (mature columella).

### 4.2 Confocal microscopy

Laser ablations were performed using a 2-photon laser (double check these specs). A 2 dimensional ROI was specified using the Zeiss ROI manager in the Zen Acquisition Black software with the time series, bleaching, and ROI modes enabled. This ROI targeted a transverse section of the root that was positioned approximately 10–20 microns shootward of the QC that spanned the entire lateral dimension of the root and spanned approximately 5–10 in the proximal/distal dimension. The ablation laser was used at 710 nm at 100 percent power for 15 iterations. In order to ensure sufficient tissue damage was achieved to induce the root to establish a new meristem, the ablation was performed in 3 Z planes: (1) in the medial plane, (2) closer to the cover slip and targeting the epidermis and cortex, and (3) further from the cover slip than the median plane and as deep as the confocal microscope could image into the tissue before imaging quality degraded. Each ablation was performed as part of a time lapse acquisition, in which typically two frames were acquired, followed by the ablation, and then three additional frames were acquired. These frames were set to be acquired 1 millisecond apart, which functionally resulted in continuous acquisitions and total time lapses of approximately 90 seconds. For 30 minute long time lapses taken on the Zeiss airyscan confocal, frames were acquired in one Z plane three minutes apart.

### 4.3 Light Sheet Microscopy

Samples were mounted for light sheet microscopy as follows: plants were grown vertically on 1/2 MS plates for 6 days. On day 6, 5 mL of 1/2 MS with 2% low melt agarose was cast into imaging cuvettes (CellVis product number #C1–1.5H-N) after being filtered through a 0.45 micron nylon filter to remove any particulates that might disturb the path of the light sheet to prepare media “blankets”. These blankets were stored at 4 degrees celsius for at least four hours prior to mounting to ensure they had fully polymerized. A sterile scalpel and forceps were used to remove a small amount of media from one end of the cuvette to create a gap that could be used to lift the media out of the cuvette. The scalpel was then gently run along the edge of the imaging chamber to free the blanket while producing minimal distortions to the media. Sterile canted forceps were then used to gently lift the media blanket out of the cuvette and placed in a sterile petri dish. Several 6 DPG seedlings were placed on top of the media blanket such that the roots were in contact with the blanket and the shoots hung off the edge. A fresh cuvette was then lowered over the blanket until the blanket made contact with the cover slip at the bottom of the cuvette. Seedlings were inspected for tissue damage under a brightfield microscope and any gaps between the blanket and the wall of the cuvette were filled in with additional filtered media prepared as above to ensure the light sheet did not pass through any air gaps. The assembled cuvettes were then placed into a growth chamber overnight oriented such that the roots pointed downward to allow the plants to recover from the stress of the mounting procedure. Roots were then imaged at 7 DPG.

Plants were stained with blue CMAC by mounting in imaging cuvettes as described above using media supplemented with blue CMAC (ThermoFisher #C2110) to 10 μM once the media had equilibrated to 30 degrees celsius. Media was then split into a number of batches equal to the number of treatment conditions to ensure that all conditions received the same concentration of blue CMAC. Additional treatments were then supplemented into the relevant batch of media as required. 5 mL of each media condition was then added to its own cuvette. and then cured for at least four hours at 4 degrees celsius. Then plants were transferred to an imaging cuvette and allowed to recover in the growth chamber overnight.

Seedlings were imaged on an inverted Leica model DMi8 outfitted with a Tilt Light Sheet Imaging System (Mizar) with filters optimized to visualization of YFP, CFP, and mCherry (Chroma). Roots were imaged with a 40X water immersion objective, with stacks spanning the entire Z dimension spaced 1.5 (double check) microns apart acquired every ten minutes in mCherry, CFP, and YFP to create time lapse movies of PlaCCI. Laser power and acquisition time was adjusted for each experiment to account for variable distance of the sample to the side of the cuvette through which the light sheet enters. A sample binning of 2 was used to improve signal brightness. For imaging of the F3 progeny of PlaCCI crossed to the WIP4 transcriptional reporter in which both transgene had been screened for stable brightness, a fourth channel - GFP - was imaged. No photobleaching was observed using these imaging conditions over the course of a time lapse. To maintain imaging quality, water was added to the 40X objective after 7–10 hours of imaging depending on the ambient humidity. This was accomplished by briefly removing the imaging cuvette between acquisitions, adding additional water to the objective, and then replacing the cuvette. The stage was adjusted to recenter the sample and then the image was realigned *post hoc* using Imaris to account for any subtle shifts in sample position. This allowed us to avoid moving the stage, which would necessitate adjusting the focus of the light sheet midway through the time lapse acquisition.

### 4.4 Single Cell RNA-seq

Protoplasts were generated as follows: To collect roots enriched for different phases of the cell cycle, root tips were synchronized with 2mM HU media as described above. To process cells synchronized in different phases in parallel, seedlings were transferred to HU media in a staggered manner such that they would be ready for harvesting at the same time.

The distal-most 400 μm of approximately 500 root tips were excised from 7 DPG seedlings and then collected via capillary action with a P200 pipette tip containing 25 μL of protoplasting buffer. These root tips were then dispensed into protoplasting solution. Root tips were gently agitated on an orbital shaker for approximately 1 hour and were gently pipetted up and down with a P1000 pipette every ten minutes after the first half hour of incubation. Root tips were then passed through a 40 micron cell strainer and any large aggregates of cells were gently pressed against the strainer using sterile flat forceps to release any cells that had so far failed to dissociate.

10X libraries were prepared from protoplasts to generate single cell RNA-seq libraries using the Chromium Next GEM Single Cell 3’ Reagent Kit v3.1 from 10X genomics following manufacturer’s instructions.

The cDNA and sequencing library fragment sizes were both measured with the Agilent Tapestation 4200 using the high sensitivity 1000 and 5000 reagents respectively. Sample concentration was detected using the Qubit HS dsDNA assay following manufacturer’s instructions. Libraries quantitation for pooling was performed as follows: the fragment size and concentration of the library in ng/μL were used to determine the molarity of the libraries with the following equation: [Lib Conc (ng/μL)]/[(Frag Length (bp) * 607.4)+157.9] * 1000000. Libraries were then diluted to 3 nM concentration and pooled for sequencing. Samples were sequenced on a Novaseq 6000 using an SP flowcell in 28×91 paired end 100 cycle mode with V1.5 reagents (100 cycles).

### 4.5 Bulk RNA-seq

For bulk RNA-seq total RNA was extracted using the Qiagen RNA micro kit following manufacturer’s instructions from sorted protoplasts. RNA quality was determined using RNA high sensitivity reagents for the Agilent Tapestation 4200. Total RNA was used to synthesize cDNA using the SMART-Seq v4 full-length transcriptome analysis kit from Takara (product # 634888) using protocol B specified in the manual on page 12. The quality of cDNA was then assessed using D1000 reagents for the Agilent Tapestation. The resulting cDNA was used to generate sequencing libraries with the Ovation Ultralow Library System V2 from Tecan (product # 0344) following manufacturer’s instructions. Libraries were then sequenced on a Novaseq 6000 with an SP flowcell in 1×100 single end 100 cycle mode with V1.5 reagents (100 cycles).

Cells were collected by FACS as follows: Root protoplasts were sorted using a BD FACS Aria II using FACS Diva software as described previously [48,49]. Briefly, protoplasts were sorted directly from protoplasting solution into a 1.5 mL microcentrifuge tube containing 350 μL of Qiagen RNA extraction buffer supplemented with beta mercaptoethanol.

Protoplasts expressing an H2B RFP fusion and a CDT1a GFP fusion under the native promoter were sorted and gated to remove doublets and debris. Then RFP positive events were identified by plotting red scale autofluorescence versus RFP and then gating for cells that showed RFP fluorescence above background as defined by a Col-0 control expressing no fluorescent proteins. In tandem, CDT1a positive cells were identified by plotting autofluorescence versus GFP and gated for GFP expression above background relative to Col-0 control. Then both the RFP+ and GFP+ populations were plotted in a histogram of RFP signal v. cell count. This revealed a population with two RFP peaks characteristic of DNA staining in dividing cells. The GFP+ population overlapped with the 2n ploidy peak, which is consistent with its expression in the G1 phase of the cell cycle and was used as a positive control. Further gates were defined based on the histogram to collect cells in G1 (2n), G2M (4n), and S (intermediate RFP signal) phases. These populations were collected simultaneously in a three way sort and the maximum number of cells were collected for each phase. This protocol was repeated independently twice to generate 6 samples for RNA-seq library preparation. Samples were snap frozen and stored at −80 degrees celsius until all samples were collected and could be processed for RNA extraction and library preparation simultaneously.

In order to use cellular ploidy as a proxy for cell cycle phase it was critical to harvest the distal-most portion of the root tip in order to avoid harvesting any cells that had already begun endoreduplication. The distal-most 200 μm of approximately 500 root tips were excised from 7 DPG seedlings and then collected via capillary action with a P200 pipette tip containing 25 μL of protoplasting buffer. These root tips were then dispensed into protoplasting solution. Root tips were gently agitated on an orbital shaker for approximately 1 hour and were gently pipetted up and down with a P1000 pipette every ten minutes after the first half hour of incubation. Root tips were then passed through a 40 micron cell strainer and any large aggregates of cells were gently pressed against the strainer using sterile flat forceps to release any cells that had so far failed to dissociate. The resulting protoplasts were then transferred to a test tube appropriate for the cell sorter and immediately processed via FACS.

### 4.6 Sequencing Data Analysis

For single cell RNA-seq the mkfastq function in cellranger 5.0.1 was used to generate fastq files from the raw sequencing output. Count matrices for single cell RNA-seq experiments were then generated with the count function and the TAIR 10.38 version of the *Arabidopsis* genome.

#### QC –

After generating count matrices using cellranger, using Seurat we filtered cells based on the number of features detected (more than 2000 and less than 10000), percent mitochondrial reads (less than 5), and total RNA molecules detected (less than 100000). This produced datasets in which the R squared coefficient between features and counts exceeded 0.93, indicating that the remaining cells in the dataset were healthy singlets. Libraries were integrated using the sctransform workflow in Seurat [50].

Cell type annotations were carried over from a control dataset that had previously been annotated based on the expression of cell type specific marker genes. Cell labels were carried over manually by examining the cluster membership of cells from the control library, which formed the same stable clusters as they had in previously when integrated with this dataset. Previous cluster identity was then manually transferred to all cells from the HU-treated datasets that shared cluster membership with the annotated cells from the control dataset.

While the single cell RNA-seq libraries were enriched for cells in each phase of the cell cycle, their cell type composition was variable. To eliminate this potential source of bias we determined the lowest number of each cell type across all enriched libraries and then randomly downsampled each cell type in each library to produce libraries with identical cell type composition. We then performed differential expression analysis with cells from each phase enriched library grouped together using Seurat’s FindAllMarkers function. Markers were ranked by percent differential expression and the top 50 for each library were chosen as cell cycle marker genes.

For cell cycle psuedotime analysis we used Monocle3 to create the UMAP embeddings with only the top 150 genes most associated with the cell cycle. We then used the learn graph and order cells functions to calculate a pseudotime trajectory for cells based on the cell cycle. To find genes that changed as a function of pseudotime we used the graph test function. We then aggregated the gene expression matrix based on evenly spaced bins along the pseudotime trajectory and clustered those bins based on gene expression to assign genes to different positions in the pseudotime trajectory.

For Bulk RNA-seq reads were trimmed using Trimmomatic version 0.39 in single end mode with the following settings: ILLUMINACLIP:TruSeq3-SE:2:30:10 LEADING:3 TRAILING:3 SLIDINGWINDOW:4:15 MINLEN:36. Trimmed reads were mapped to the Arabidopsis TAIR10 genome using HISAT2 version 2.2.1. Reads mapping to genes were counted with Rsubread (version 1.22.1) featureCounts in single end mode with a minimum overlap of 5 and counting only primary alignments and ignoring duplicates. Reads were normalized using the TPM calculation and the resulting count matrix was used to calculate mean values per condition, filtered to remove genes with low expression and low variance, and then clustered via k-means clustering. The number of k (8) was chosen to reflect the total permutations of expression changes (up or down) and cell cycle phases (G1, S, G2M).

Data visualization was generated using ggplot2 with tidyverse, seurat, pheatmap, treemap and monocle3.

### 4.7 Imaging Data Analysis

Long-term time-lapse images were registered in 3 dimensions by first detecting objects (either nuclei, WOX5, or WIP4 expression) and then using detected objects to correct the reference frame for the time lapse in 3 dimensions. The new reference frame was then used to correct the time lapse for both translational and rotational drift. Once drift corrected, nuclei were then segmented again using the spot detection tool. Once segmented, statistics for all nuclei were exported to R for further analysis.

For still images 3 dimensional segmentation was performed in trackmate by treating the Z dimension as a time dimension. Nuclei were segmented based on the mCherry channel and then data for each channel within nuclei was exported to R for further analysis.

Confocal image stacks were taken such that nuclei would appear in at least two consecutive slices. Therefore all nuclei that appeared in only one slice were discarded. For the remaining nuclei, Blue CMAC signal was scaled from 0 to 1 per cell file to render them comparable. In the case of short term time lapses of PlaCCI roots stained with Blue CMAC taken using confocal microscopy, drift was corrected in 2 dimensions using the correct 3D drift plugin in FIJI prior to trackmate segmentation. Nuclei were filtered if they were not tracked for the entire time lapse. Then Blue CMAC signal was calculated as a change over the value at time zero.

### 4.8 In Situ Hybridization

#### Probe selection –

Candidate probes were selected from the top marker set described above if they had a were expressed in at least 80 percent of cells from the target phase and if they exceeded a differential expression threshold of 0.25 LFC based on a differential expression test performed in seurat with the design. Then the average expression for each gene in the marker set within a given phase was calculated. The top 5 most highly expressed genes from each phase that had passed the differential expression filtering step were chosen as candidates for individual interrogation. The expression of this small set of genes was examined manually to ensure there was no cell-type-specific bias. Finally the most strongly expressed candidates from this set were chosen for probe design. We prioritized genes from these sets that had either unknown function or were not previously characterized as being cell cycle regulated. Probe design was performed by Molecular Instruments. *In situ* hybridization was performed as described previously [51] with minor modifications.

## Supplementary Material

Supplement 1

Supplement 2

Supplement 3

## Figures and Tables

**Table 1:** Your Table Caption

Condition	Rep	Movie Duration	G1 end observed?	Median G1 Duration (h)	Number of events	Median G1 Duration by condition (h)	Median max G1 Duration by condition (h)	Median Duration Median Duration Fraction (h in G1/total movie length)
Ablation	1	12	No	7.33	17	3.7	3.7	0.3
			Yes	1.83	25			
Ablation	2	12.8	No	5.17	6	3.3	3.3	0.3
			Yes	2	12			
Control	1	13.3	No	10.2	15	11.5	20	0.9
			Yes	6	7			
Control	2	8.3	No	6.83	11	6.83	20	0.8
			Yes					
