# Supplementary material for: Glutathione accelerates the cell cycle and cellular reprogramming in plant regeneration
Source: bioRxiv. 2024 Oct 15:2023.11.28.569014. Preprint. [Version 4] doi: 10.1101/2023.11.28.569014 (PMC10760015; doi:10.1101/2023.11.28.569014)
Supplement: Supplement 6 [file NIHPP2023.11.28.569014v4-supplement-6.pdf]

bioRxiv preprint doi: https://doi.org/10.1101/2023.11.28.569014; this version posted October 15, 2024. The copyright holder for this preprint (which was not certified by peer review) is the author/funder, who has granted bioRxiv a license to display the preprint in perpetuity. It is made available under aCC-BY 4.0 International license.

Supplemental Figure Titles and Legends

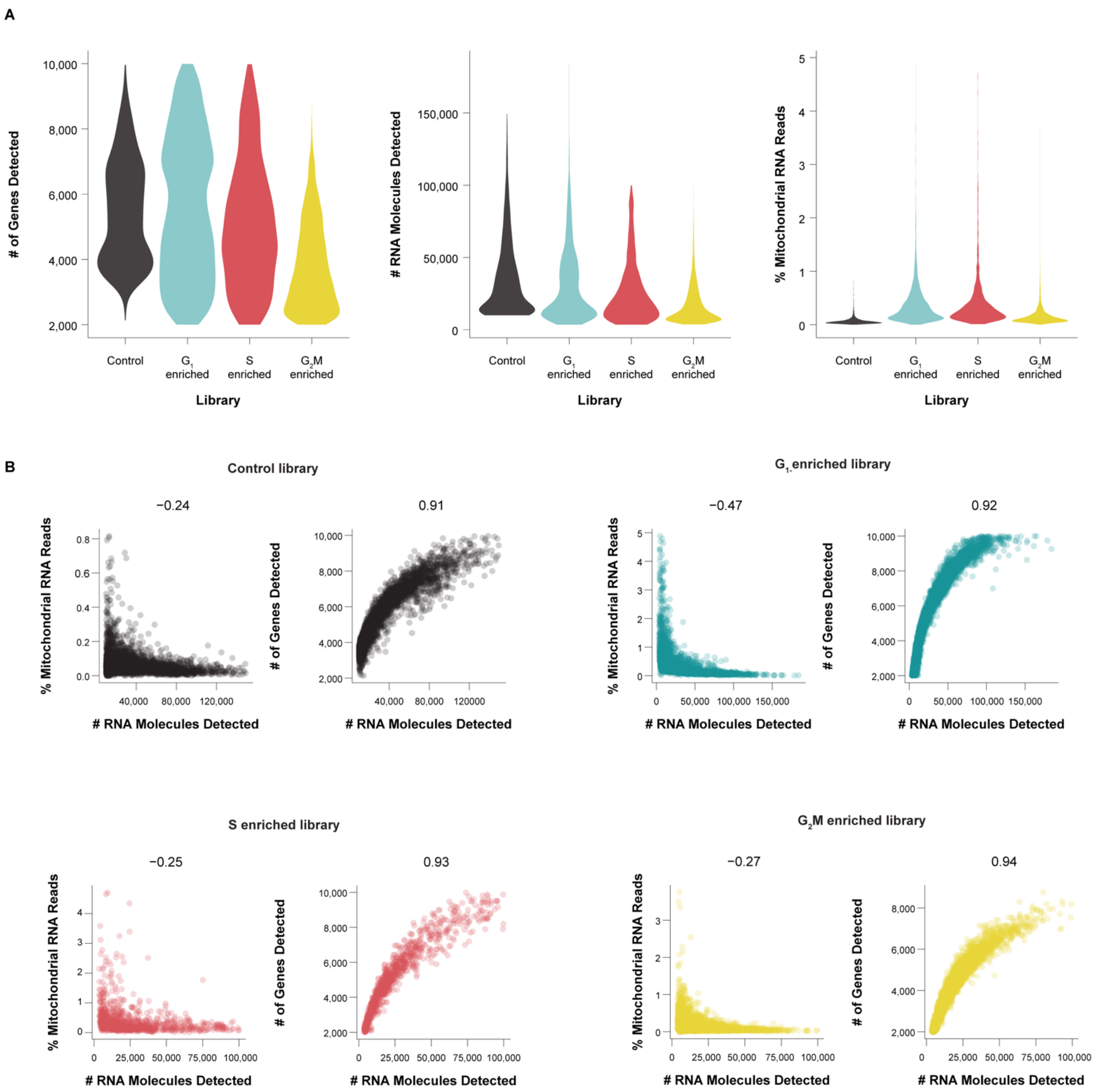

<u>Figure S1</u>. Single Cell RNA-seq Profiles Show Robust Signals in Quality Control; Related to Figure 1. (A) Violin plots showing the number of genes, RNA molecules, and the percentage of reads from mitochondrial genes, per cell in each scRNA-seq library. (B) For each library, a pair of scatter plots shows (1) the anti-correlation between percent mitochondrial reads and number of RNA molecules detected (at left), and (2) the correlation between the number of genes and the number of unique RNA molecules detected (at right). Correlation coefficient is shown above the plot.

bioRxiv preprint doi: https://doi.org/10.1101/2023.11.28.569014; this version posted October 15, 2024. The copyright holder for this preprint (which was not certified by peer review) is the author/funder, who has granted bioRxiv a license to display the preprint in perpetuity. It is made available under aCC-BY 4.0 International license.

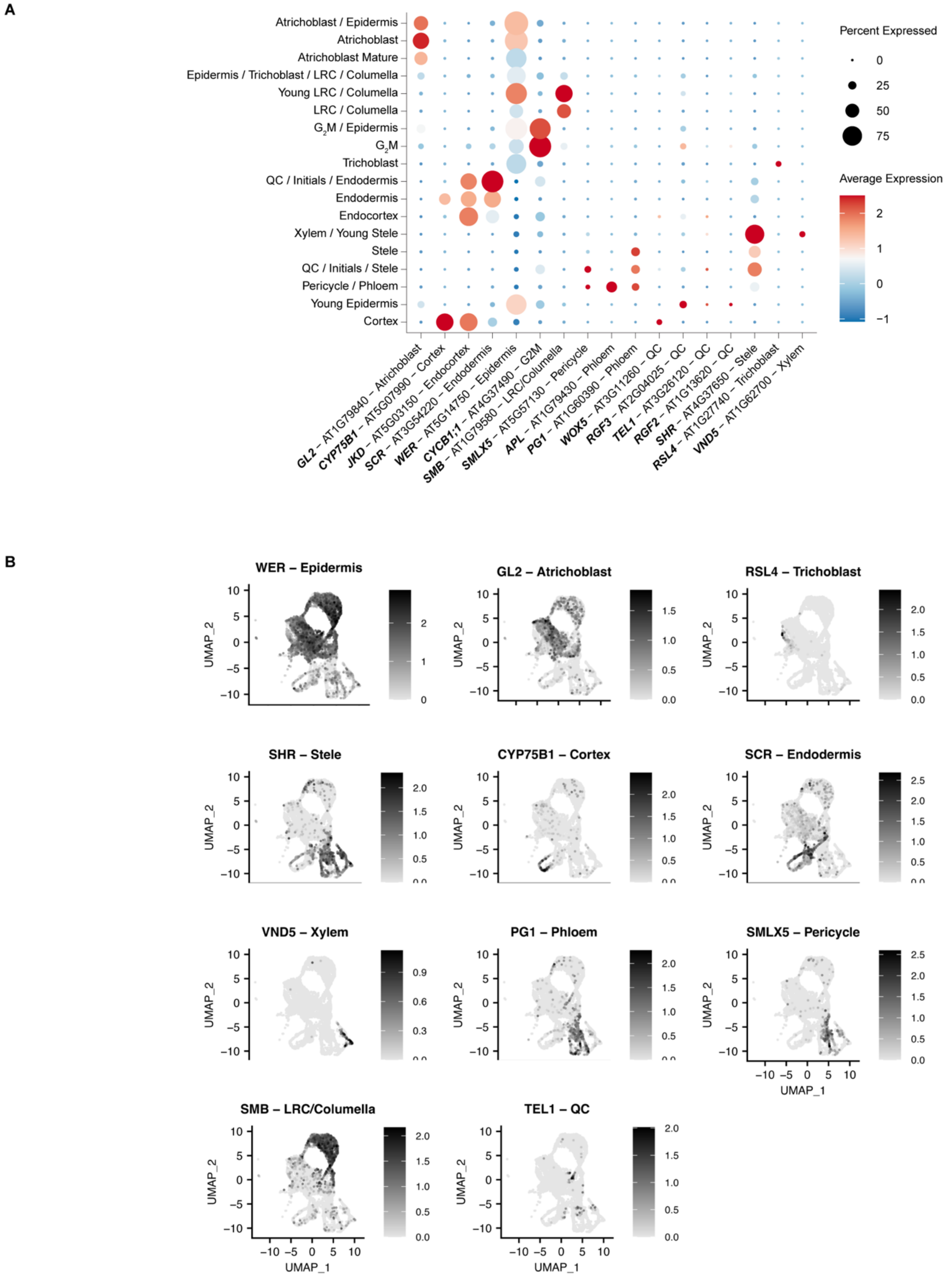

<u>Figure S2</u>: Markers robustly identify cell types in phase-enriched libraries, Related to Figure 1. (A) A dot plot showing the expression of marker genes across clusters defined by cell type in the integrated phase-enriched libraries. Size of the dot shows the percentage of cells in a cluster expressing the marker and the colormap shows the average expression of the marker in the cluster. (B) UMAPs highlighting the highly localized expression of various cell-type specific marker genes, as expected for robust capture of cell identities

bioRxiv preprint doi: https://doi.org/10.1101/2023.11.28.569014; this version posted October 15, 2024. The copyright holder for this preprint (which was not certified by peer review) is the author/funder, who has granted bioRxiv a license to display the preprint in perpetuity. It is made available under aCC-BY 4.0 International license.

in scRNA-seq profiles. The cells are not grouped by phase and demonstrate the overall quality of the cells in the ability to capture clusters with clear cell identity.

bioRxiv preprint doi: https://doi.org/10.1101/2023.11.28.569014; this version posted October 15, 2024. The copyright holder for this preprint (which was not certified by peer review) is the author/funder, who has granted bioRxiv a license to display the preprint in perpetuity. It is made available under aCC-BY 4.0 International license.

A

	G_1 enriched	S enriched	G_2M enriched
Atrichoblast	74	74	74
Atrichoblast / Epidermis	15	15	15
Atrichoblast Mature	20	20	20
Cortex	12	12	12
Endocortex	14	14	14
Endodermis	3	3	3
Epidermis / Trichoblast / LRC / Columella	89	89	89
G_2M	128	37	554
G_2M / Epidermis	8	8	8
LRC / Columella	186	186	186
Pericycle / Phloem	28	28	28
QC / Initials / Endodermis	18	18	18
QC / Initials / Stele	115	115	115
Stele	23	23	23
Trichoblast	45	45	45
Xylem / Young Stele	36	36	36
Young LRC / Columella	169	169	169
Young Epidermis	112	112	112

B

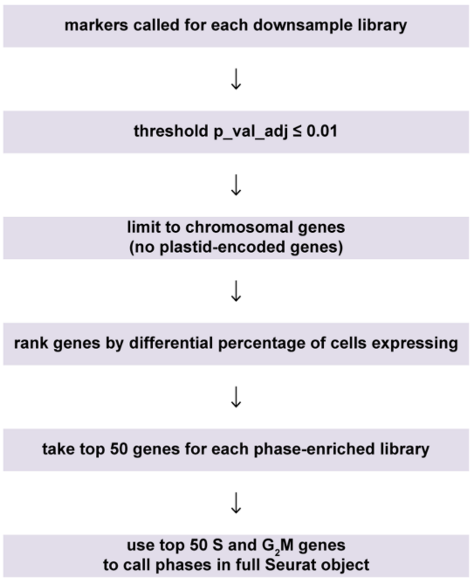

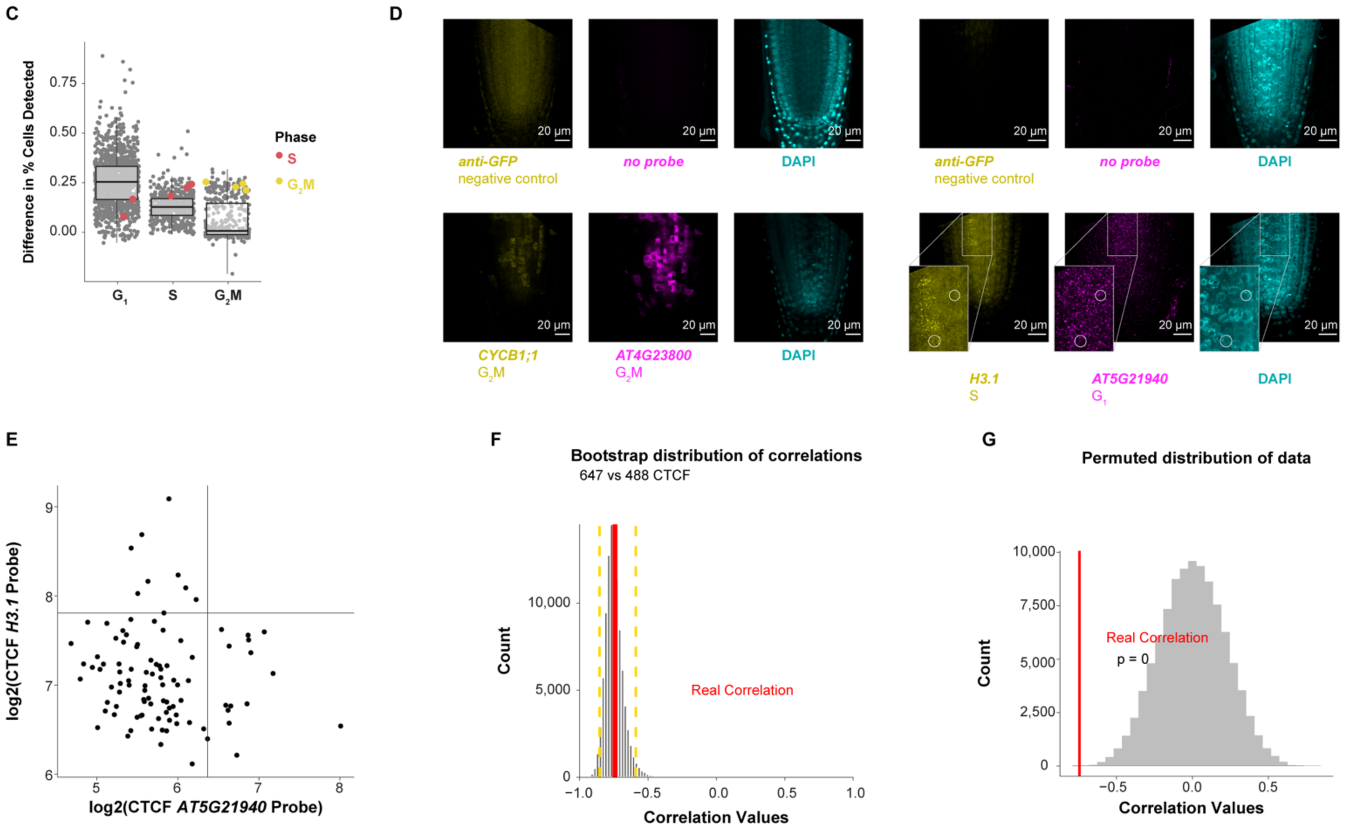

<u>Figure S3</u>. Data analysis methods identify cell phase markers with *in situ* validation of a new G1 marker; Related to Figure 1. (A) Cell counts for down-sampled phase-enriched libraries, ensuring each cell type contributed an equal number of cells to each phase enrichment analysis and each cell type contributed to phase enrichment analysis. (B) Differential expression analysis pipeline to identify phase markers. (C) Genes (each dot) categorized as differentially expressed in specific phase-synchronized libraries. The y axis

bioRxiv preprint doi: https://doi.org/10.1101/2023.11.28.569014; this version posted October 15, 2024. The copyright holder for this preprint (which was not certified by peer review) is the author/funder, who has granted bioRxiv a license to display the preprint in perpetuity. It is made available under aCC-BY 4.0 International license.

represents the difference in the percentage of cells in which the gene is expressed in target versus non-target libraries. The highlighted genes are gold standard markers of phase-specific expression, showing high expression in many cells in the appropriate phase-synchronized library (x axis categories). (D) Representative images of G2M (left) and S phase (right) from *in situ* experiments shown with their corresponding negative controls as annotated. Insets highlight examples of cells where G1 and S probe signal is anti-correlated, which is quantified in the next panel. (E) Anti-correlation with signal cutoffs shown for H3.1 and AT5G21940 probes with signal cutoffs determined empirically via change point analysis[83]. Values come from three root median sections in which all cells were hand segmented based on DAPI counterstain. (F) Bootstrap distribution of correlation values between H3.1 and AT5G21940 probe signals shows the determined anti-correlation falls within the 95% confidence interval (yellow dotted lines). (G) Permutation distribution of the correlation between H3.1 and AT5G21940 probe signals shows the actual anti-correlation falls well outside of the null distribution (pvalue = 0).

bioRxiv preprint doi: https://doi.org/10.1101/2023.11.28.569014; this version posted October 15, 2024. The copyright holder for this preprint (which was not certified by peer review) is the author/funder, who has granted bioRxiv a license to display the preprint in perpetuity. It is made available under aCC-BY 4.0 International license.

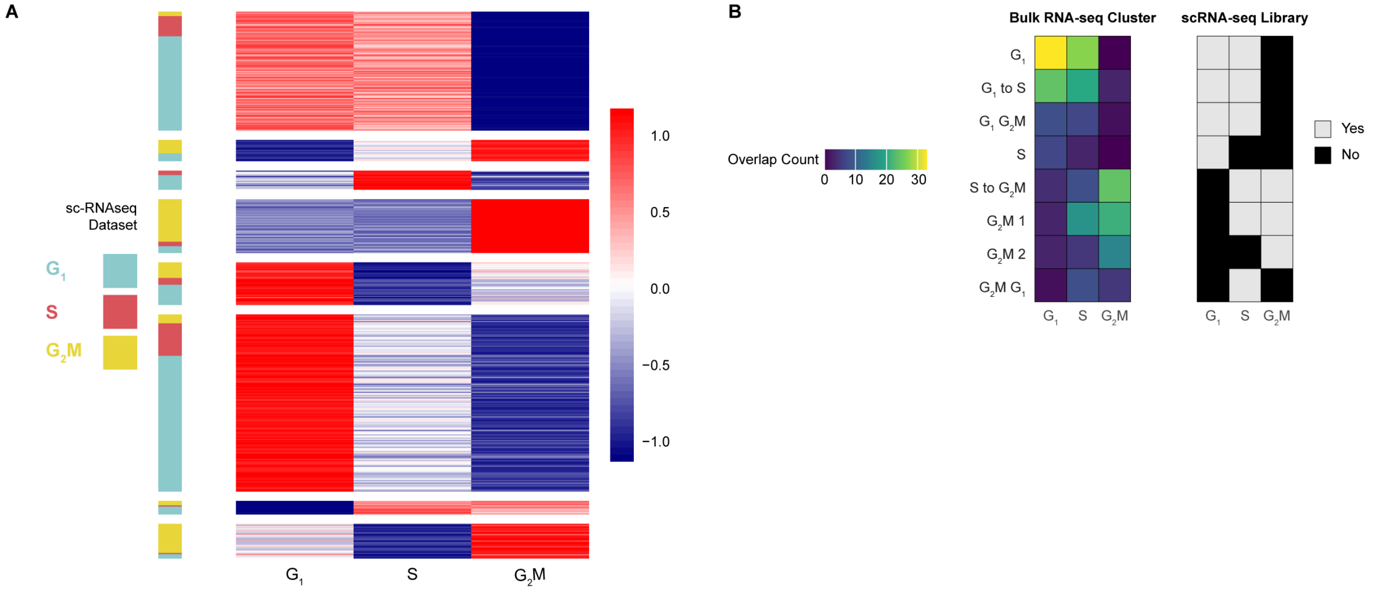

<u>Figure S4:</u> Bulk RNA-seq profiles of the cell cycle confirm phase-enriched scRNA-seq; Related to Figure 1.

(A) Gene expression heatmap (red and blue) in which each row is a gene and each column represents the average expression profile across bulk RNA-seq profiles where the three libraries represent cells sorted by ploidy level as a proxy for phase. The color bar to the left indicates the independent cell cycle phase classification of each gene from analysis of the synchronized scRNA-seq library. In the bulk RNA-seq analysis, genes were grouped into 8 k-means clusters. Agreement between the two independent analyses is indicated by groups of genes showing a sc-RNA-seq classification and enrichment in the appropriate ploidy sorted cell library. Strong agreement is shown for G1 and G2/M, while S-phase is not well defined in the ploidy sorting (B) Heatmaps showing the number of overlapping genes (left) and the statistical significance of the overlap (right) between differentially expressed genes from phase-enriched scRNA-seq (columns) and gene expression clusters of ploidy-sorted cells determined by k-means clustering (rows). Yes=statistically significant overlap at $p<0.05$ by Fishers exact test. See also Table S4.

bioRxiv preprint doi: https://doi.org/10.1101/2023.11.28.569014; this version posted October 15, 2024. The copyright holder for this preprint (which was not certified by peer review) is the author/funder, who has granted bioRxiv a license to display the preprint in perpetuity. It is made available under aCC-BY 4.0 International license.

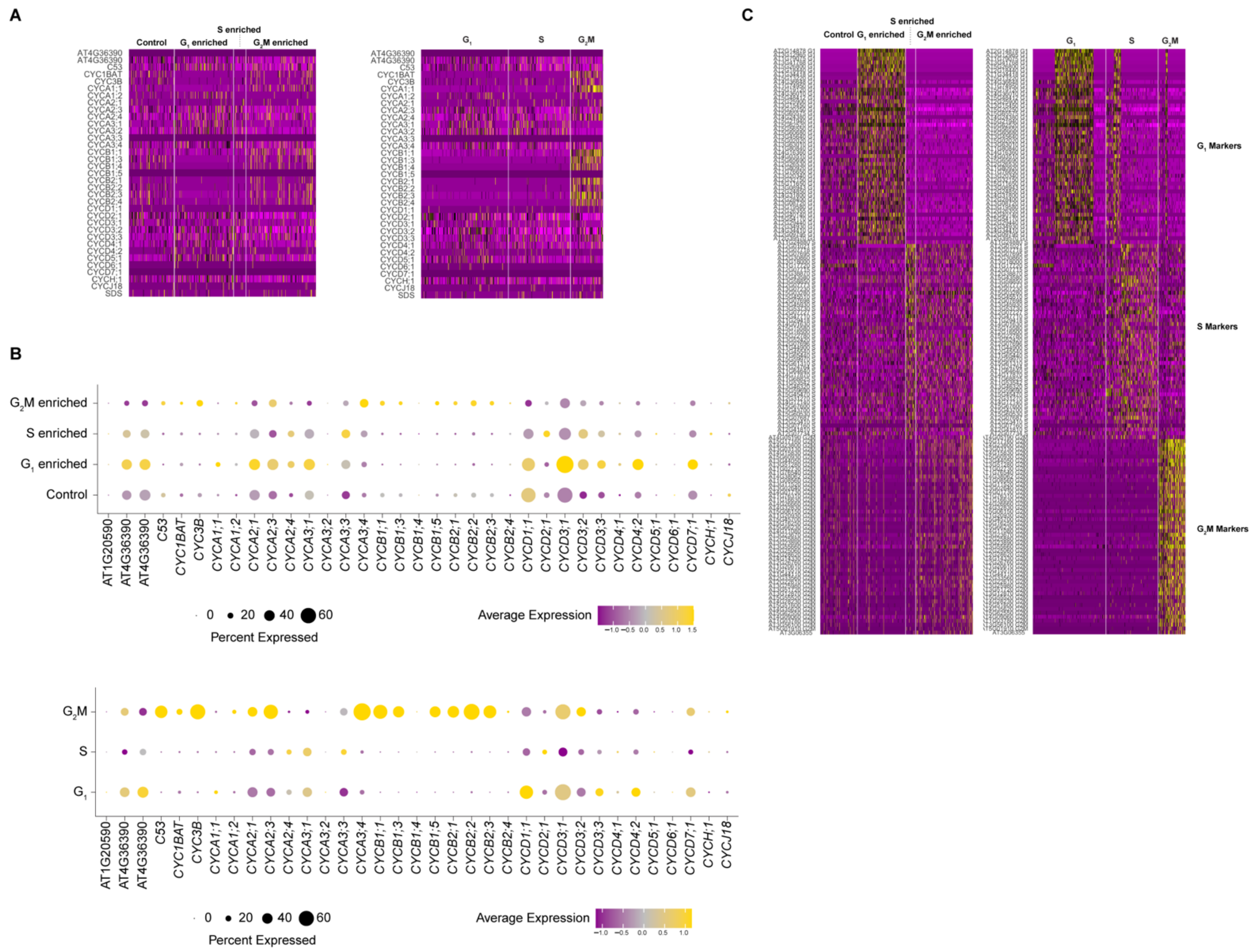

Figure S5: Enrichment analysis for phase markers shows agreement with known cell cycle markers but identifies more robust markers; Related to Figure 1. (A) Heatmaps comparing expression of classical cell-cycle markers (rows) in cells (columns) grouped by the phase enrichment library from which they came (left), which may still contain cells from a mixture of phases, vs. cells assigned to phase based on marker analysis (right). At left, some enrichment of markers is visible but phase enriched libraries still contain cells in the non-target phase. At right, enrichment of known markers is more prominent when cells are assigned to phase by our analysis pipeline, which is independent of the expression of the classical cell cycle markers. (B) A summary analysis of the heatmap data in A. Dotplots show the expression of cyclins in phase-enriched libraries (top) vs phases assigned with our top marker genes (bottom). Cyclins are expressed in the appropriate datasets despite their sparseness (top). Cyclin expression behaves well based on phase assignments performed with our marker genes (bottom). (C) Following the same comparison as in A with the top 50 markers assigned by our pipeline. At left, the markers are shown based on their enrichments in the different phase libraries. These agree with classical markers but the analysis shows the new markers have higher expression and are more frequently detected in single-cell profiles. At right, the analysis show cells classified by phase using the top 50 markers. Note that many G1-phase markers also express in early S phase, but S-phase has distinct markers to separate G1 and early S.

bioRxiv preprint doi: https://doi.org/10.1101/2023.11.28.569014; this version posted October 15, 2024. The copyright holder for this preprint (which was not certified by peer review) is the author/funder, who has granted bioRxiv a license to display the preprint in perpetuity. It is made available under aCC-BY 4.0 International license.

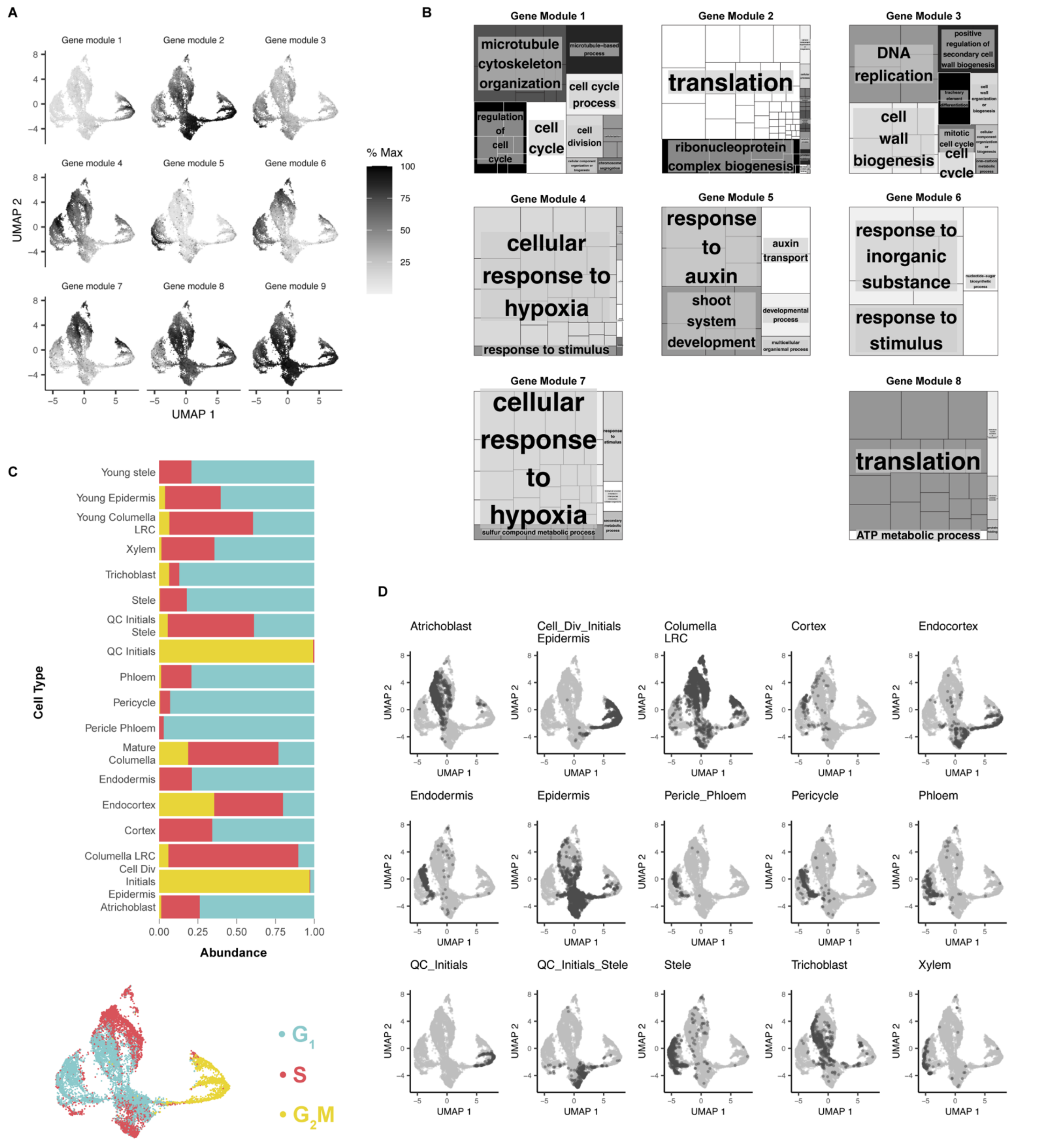

Figure S6. Cells of the same identity group together even when clustered by only cell cycle markers; Related to Figure 2. (A) Analysis of gene modules that are preferentially expressed along the cell-cycle pseudotime ordering, as determined by Monocle3 (see Methods). Grayscale shows the aggregate gene expression of each gene group. (B) GO-terms associated with the corresponding gene group shown in B. No significant GO terms were found for gene module 8. (C) Relative abundances of phases among each cell type are shown. (D) UMAP outputs of pseudotime analysis clustered using the top 50 cell-cycle markers with an

bioRxiv preprint doi: https://doi.org/10.1101/2023.11.28.569014; this version posted October 15, 2024. The copyright holder for this preprint (which was not certified by peer review) is the author/funder, who has granted bioRxiv a license to display the preprint in perpetuity. It is made available under aCC-BY 4.0 International license.

independent analysis of cell identity mapped onto the UMAP trajectories. In each panel, a different cell type is highlighted in red. At left, a key shows the cell cycle classification for each cluster in the UMAP.

bioRxiv preprint doi: https://doi.org/10.1101/2023.11.28.569014; this version posted October 15, 2024. The copyright holder for this preprint (which was not certified by peer review) is the author/funder, who has granted bioRxiv a license to display the preprint in perpetuity. It is made available under aCC-BY 4.0 International license.

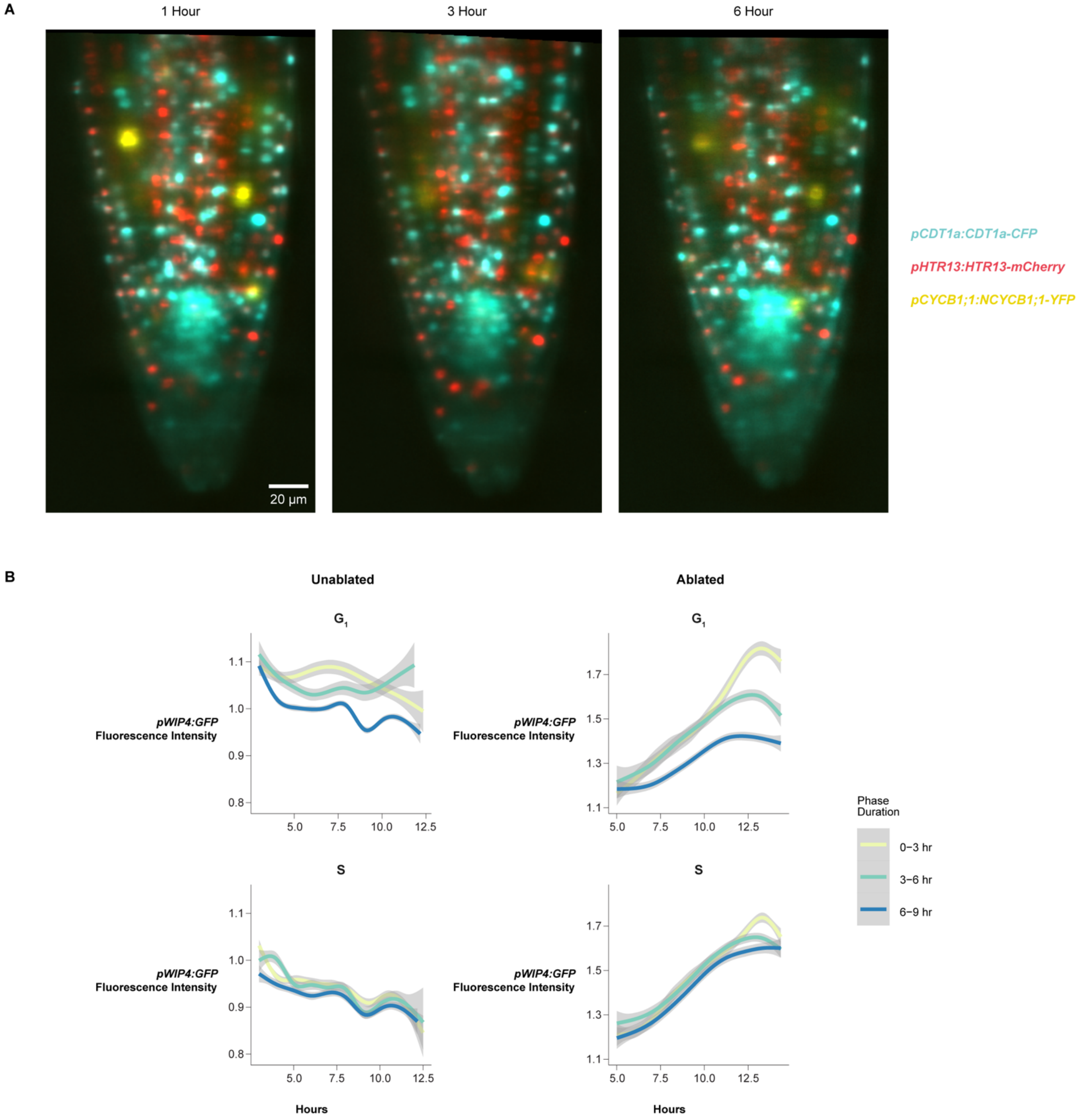

<u>Figure S7:</u> The appearance of newly reprogrammed cell identity correlates with rapid G1 phases; Related to Figure 3. (A) Representative images of a control root expressing PlaCCI and *pWIP4:GFP* at 1, 3, and 6 hr time points during a time-lapse acquisition, showing consistent distribution of each of the three markers over time under imaging conditions (B) Quantification of the WIP4 signal intensity in G1 phase and S phase cells over the duration of time-lapse movies. The figure represents the complete analysis of data shown in Figure 3E.

bioRxiv preprint doi: https://doi.org/10.1101/2023.11.28.569014; this version posted October 15, 2024. The copyright holder for this preprint (which was not certified by peer review) is the author/funder, who has granted bioRxiv a license to display the preprint in perpetuity. It is made available under aCC-BY 4.0 International license.

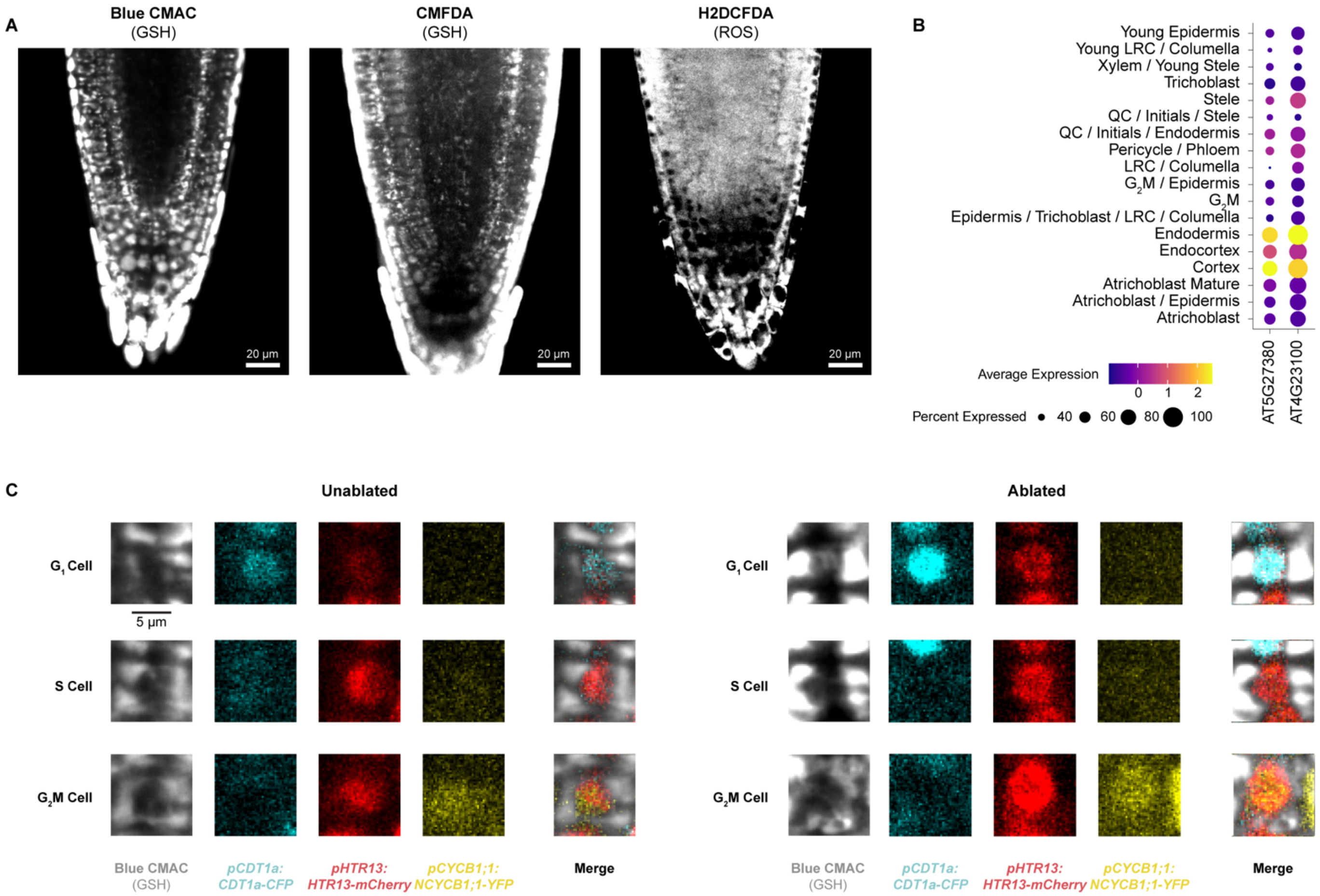

<u>Figure S8.</u> ROS and GSH dyes show different tissue localization patterns; Related to Figure 4. (A) Representative confocal microscopy images of seedlings stained for GSH (Blue CMAC, CMFDA) or ROS (H2DCFDA) under control conditions. Note that the two GSH dyes agree and show prominent ground tissue staining. Note that CMFDA and H2DCFDA, with similar chemical structure but different target molecules, show different staining patterns. (B) Expression of GSH1 and GSH2 represented as dot plot derived from scRNA-seq profiles in different root cell types. Note the prominent expression in endodermis and cortex, in agreement with the GSH dyes. (C) PlaCCI signal at time 0 for cells shown in Figure 4D, which demonstrates a GSH burst in G1 nuclei prior to fast divisions. Exogenous application of GSH did not cause a shift in the number of G1 cells (root n = 37, nuclei n = 9100, no significant difference between treatment and control by student’s t test).

bioRxiv preprint doi: https://doi.org/10.1101/2023.11.28.569014; this version posted October 15, 2024. The copyright holder for this preprint (which was not certified by peer review) is the author/funder, who has granted bioRxiv a license to display the preprint in perpetuity. It is made available under aCC-BY 4.0 International license.

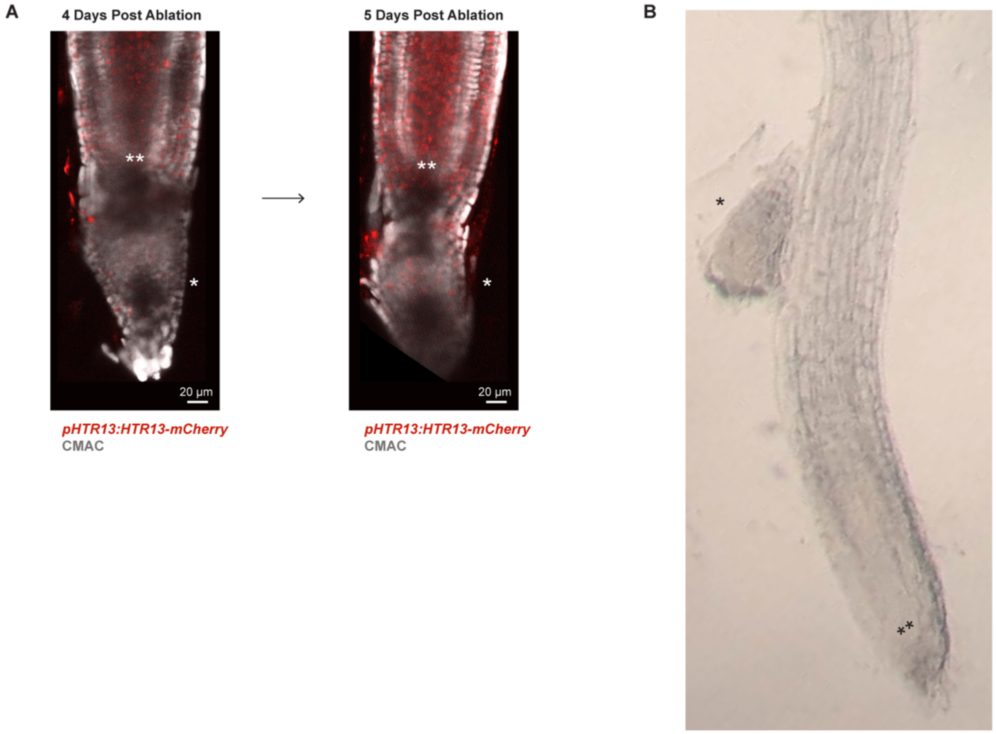

<u>Figure S9</u>. Transverse ablation leads to the reformation of a new root tip similar to the root tip excision procedure; Related to Figure 4. (A) Representative confocal images of seedlings (grown on standard ½ MS and then mounted in an imaging cuvette) undergoing regeneration. Between days 4 and 5 post ablation it becomes apparent that new columella above the ablation is established proximal (shootward) to the original QC (*), which is below the ablation. The tapered root cap, which includes the columella, is apparent distal to the new QC (**), both of which are above the ablation site. (B) At a later time point, the original root tip (*) is sloughed off as growth continues from the new QC/stem cell niche (**) in the same seedling shown in the lower panel of A.

bioRxiv preprint doi: https://doi.org/10.1101/2023.11.28.569014; this version posted October 15, 2024. The copyright holder for this preprint (which was not certified by peer review) is the author/funder, who has granted bioRxiv a license to display the preprint in perpetuity. It is made available under aCC-BY 4.0 International license.

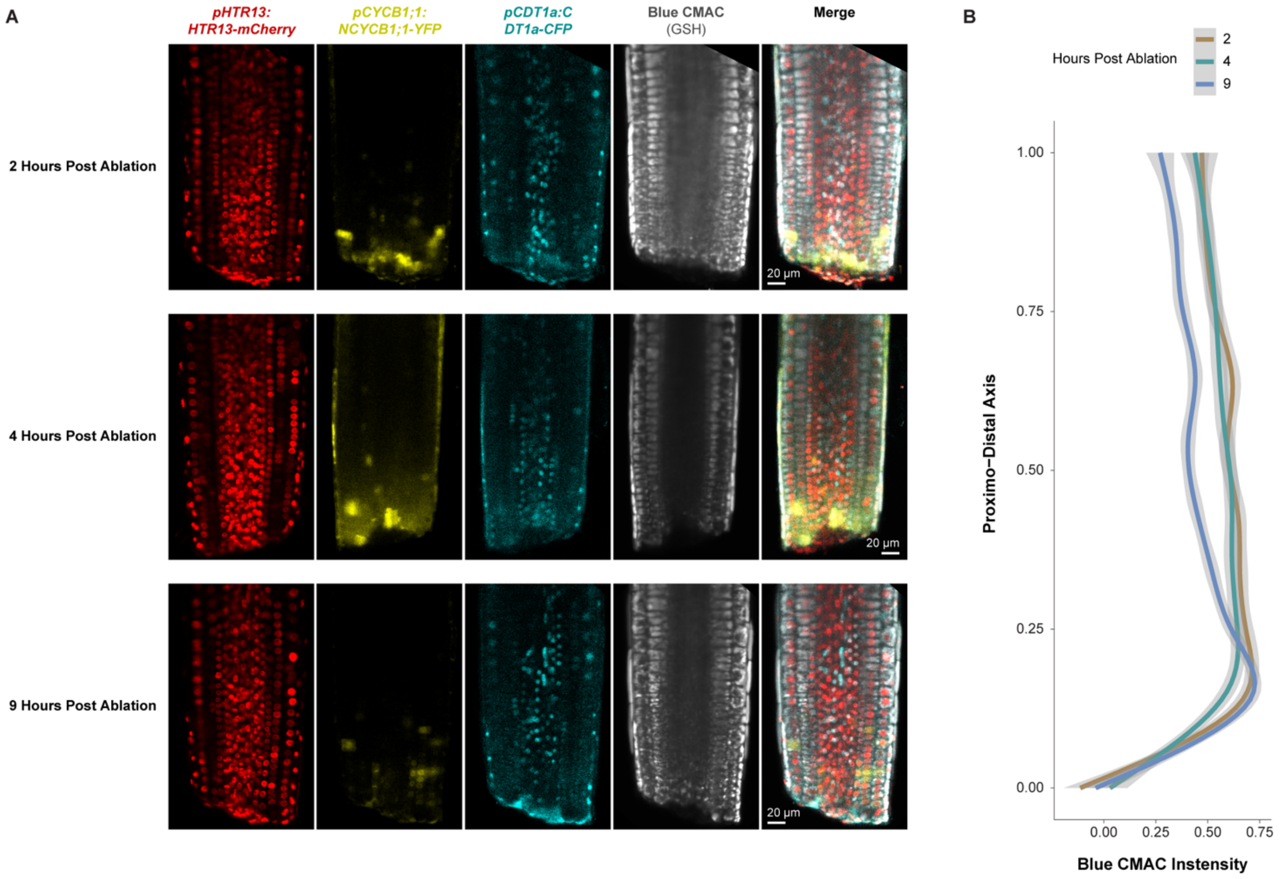

<u>Figure S10.</u> GSH dye CMAC is brightest in the same region where cells undergo rapid division and shortened G1 during regeneration; related to Figure 4. (A) Representative confocal images of PlaCCI roots stained with Blue CMAC. Images were taken 2, 4, and 9 hpc. (B) Quantification of nuclear CMAC staining intensity along the proximal-distal axis at different time points after ablation. The y-intercept represents the ablation site and the range of the y-axis represents the visible length of root imaged in the frame as shown in A. Note the peak of CMAC intensity right above the cut site between 0.00 and 0.25 on the longitudinal axis of the root (y-axis), which is highest at 2-4 hr post cut and begins to dissipate above point 0.25 at 9 hr.

bioRxiv preprint doi: https://doi.org/10.1101/2023.11.28.569014; this version posted October 15, 2024. The copyright holder for this preprint (which was not certified by peer review) is the author/funder, who has granted bioRxiv a license to display the preprint in perpetuity. It is made available under aCC-BY 4.0 International license.

Supplemental Table Titles and Legends

Table S1. Summary of all differentially regulated genes identified in this study; Related to Figure 1 and S1-S5. KmeansClust refers the cluster identified in S4. Sequencing method indicates which method the gene was detected in. sc_log2foldchange refers the log2 fold change in the scRNA-seq phase marker identification analysis. Similarly, sc_pval_adj, sc_phase, and sc_diffpct refer to the adjust p-value, enriched library, and difference in percent cells expressing in the same analysis. Marker indicates which phase a gene was identified to be a marker of for the top 50 markers. Permissive marker is the same, but includes the top 200 markers.

Table S2. Gold standard markers from prior transcriptional studies; Related to Figure 1.

Table S3. Gene Set Enrichment analysis results for the top 50 and top 200 marker sets as well as the G1 bulk RNAseq clusters; Related to Figures 1 and S4.

Table S4. Differential expression analysis of G1 subpopulations; Related to Figure 2. In the column titled “cell_group”, left refers genes with upregulated expression in the leftmost branch of cells shown in Figure 2C and upper refers to genes with upregulated expression in the uppermost branch of cells shown in Figure 2C.

Table S5. G1 duration summary; Related to Figure 3.

Table S6. Cell type specific phase marker matrix. Related to Figure 2. Each column represents a cell type plus a cell cycle phase category. Each row represents a gene. A value of 1 indicates a given gene (row) is a marker for a phase in a particular cell type (column). A value of 0 indicates a gene is not a marker of a cell cycle plus phase type.

Supplemental Movie Titles and Legends

Movie S1. Time lapse movie showing G2/M duration during homeostatic growth; Related to Figure 2. A median section from a time lapse is shown for the *pCYCB1;1:NCYCB1;1-YFP* component of the PlaCCI reporter to show the behavior of dividing cells. Time stamp is shown in days:hr:min. CYCB1;1-YFP is shown in yellow.

Movie S2. Time lapse movie showing two replicates of PlaCCI crossed to WIP4 during homeostatic growth; Related to Figure 3. Time stamp is shown in days:hr:min. *pCYCB1;1:NCYCB1;1-YFP* is shown in yellow, *pCDT1a:CDT1a-CFP* is shown in cyan and *pHTR13:HTR13-mCherry* is shown in red. The *pCDT1a:CDT1a-CFP* channel is also shown in a separate panel. Panels A and B show two replicates of time lapses taken under control conditions of roots undergoing homeostatic growth.

Movie S3. Time lapse movie showing two replicates of PlaCCI crossed to *pWIP4:GFP*, *PET111:YFP*, or *pWOX5:YFP* during regeneration; Related to Figure 3. Time stamp is shown in days:hr:min and represents time post ablation. *pCYCB1;1:NCYCB1;1-YFP* is shown in yellow, *pCDT1a:CDT1a-CFP* is shown in cyan and *pHTR13:HTR13-mCherry* is shown in red. The *pCDT1a:CDT1a-CFP* channel is also shown in a separate

bioRxiv preprint doi: https://doi.org/10.1101/2023.11.28.569014; this version posted October 15, 2024. The copyright holder for this preprint (which was not certified by peer review) is the author/funder, who has granted bioRxiv a license to display the preprint in perpetuity. It is made available under aCC-BY 4.0 International license.

panel. PlaCCI is shown in each panel and additional reporters are shown as follows: (A) *PET111:YFP*, (B) *pWOX5:YFP*, (C) *PET111:YFP*, (D) *pWIP4:GFP*. In panels A, B, and C the following reporters are shown in yellow in addition to CYCB1;1: in the top and bottom panels on the left, PET111 is shown in yellow. In the top right panel, WOX5 is shown in yellow. Panel D shows WIP4*pWIP4:GFP* expression in grayscale as a separate panel.

Movie S4. Time lapse showing GSH burst following an ablation; Related to Figure 4. Time stamp is shown in days:hr:min where 00:00:00 marks the first frame of the time lapse. *pCYCB1;1:NCYCB1;1-YFP* is shown in yellow, pCDT1a:CDT1a-CFP is shown in cyan and pHTR13:HTR13-mCherry is shown in red, and Blue CMAC is shown in grey. The Blue CMAC channel is also shown in a separate panel.
