# Supplementary material for: Glutathione accelerates the cell cycle and cellular reprogramming in plant regeneration
Source: bioRxiv. 2024 Jun 20:2023.11.28.569014. Preprint. [Version 3] doi: 10.1101/2023.11.28.569014 (PMC10760015; doi:10.1101/2023.11.28.569014)
Supplement: Supplementary file 1 [file NIHPP2023.11.28.569014V3-supplement-1.pdf]

bioRxiv preprint doi: https://doi.org/10.1101/2023.11.28.569014; this version posted June 20, 2024. The copyright holder for this preprint (which was not certified by peer review) is the author/funder, who has granted bioRxiv a license to display the preprint in perpetuity. It is made available under aCC-BY 4.0 International license.

Supplemental Figure Titles and Legends

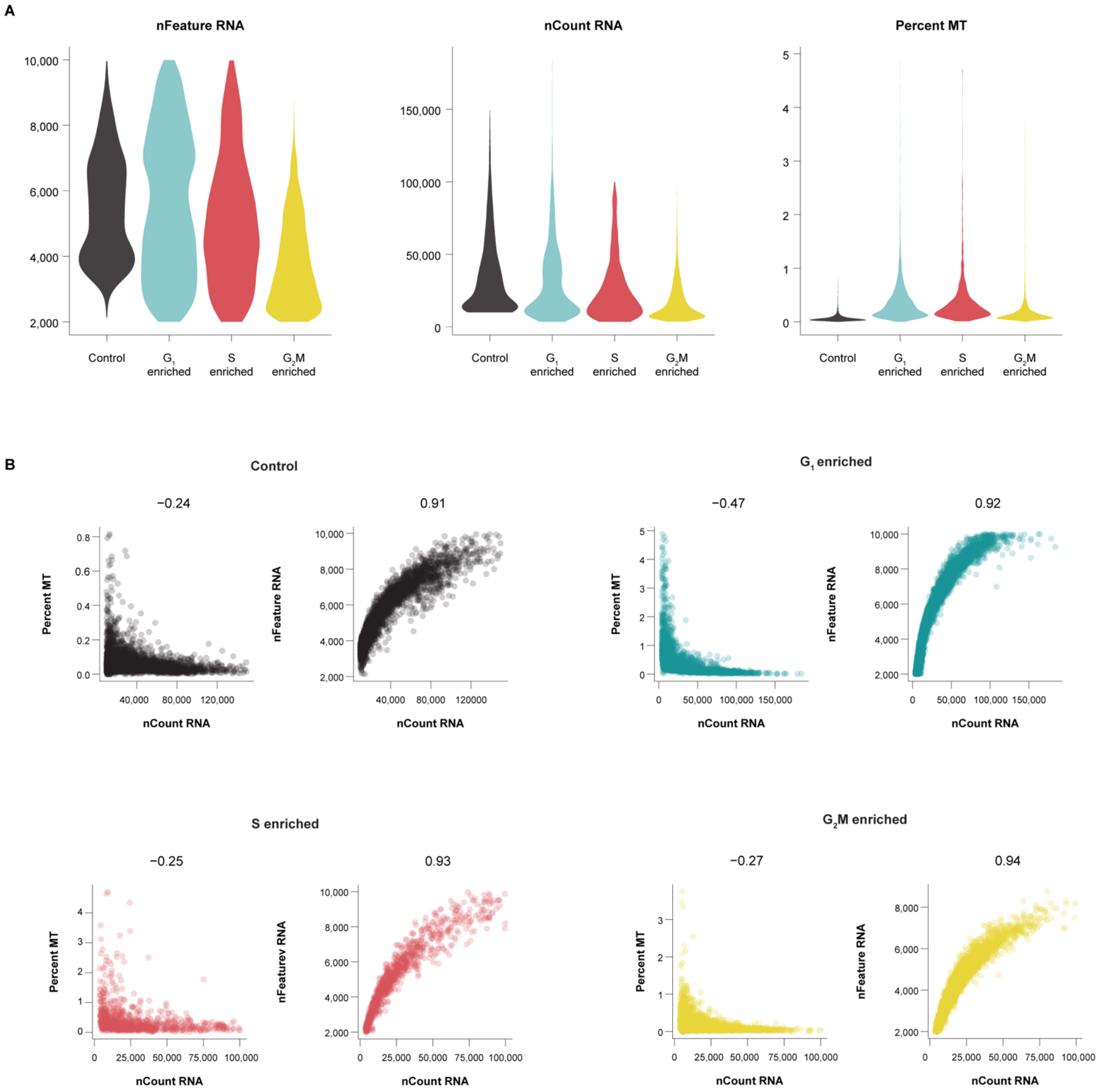

<u>Figure S1</u>. Single Cell RNA-seq Profiles Show Robust Signals in Quality Control; Related to Figure 1. (A) Violin plots showing the number of genes (nFeature_RNA), RNA molecules (nCount_RNA), and the percentage of reads from mitochondrial genes (Percent_MT) per cell in each scRNA-seq library. (B) For each library, a pair of scatter plots shows (1) the anti-correlation between percent mitochondrial reads and number of RNA molecules detected (at left), and (2) the correlation between the number of genes and the number of unique RNA molecules detected (at right). Correlation coefficient is shown above the plot.

bioRxiv preprint doi: https://doi.org/10.1101/2023.11.28.569014; this version posted June 20, 2024. The copyright holder for this preprint (which was not certified by peer review) is the author/funder, who has granted bioRxiv a license to display the preprint in perpetuity. It is made available under aCC-BY 4.0 International license.

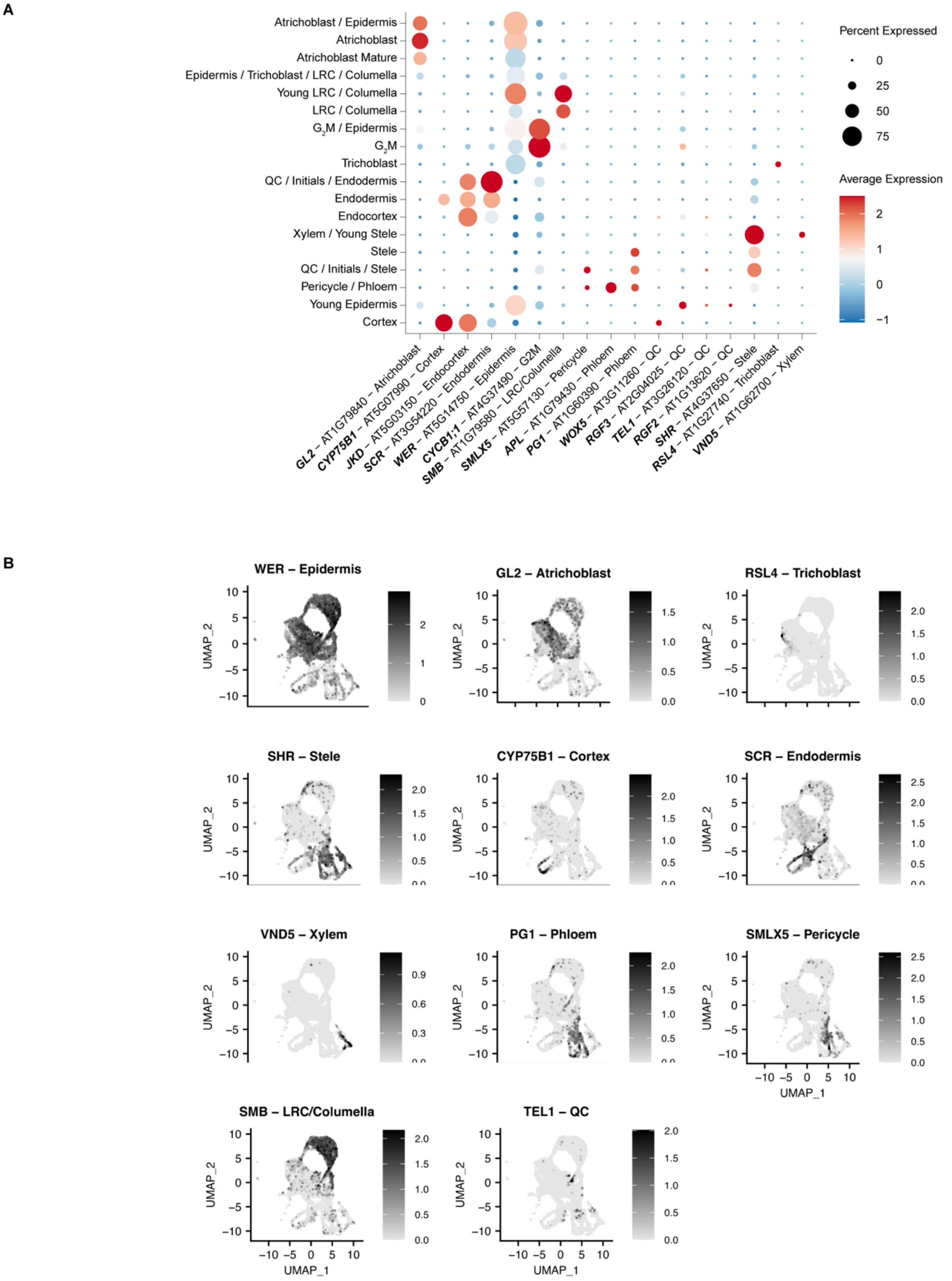

<u>Figure S2</u>: Markers robustly identify cell types in phase-enriched libraries, Related to Figure 1. (A) A dot plot showing the expression of marker genes across clusters defined by cell type in the integrated phase-enriched libraries. Size of the dot shows the percentage of cells in a cluster expressing the marker and the colormap shows the average expression of the marker in the cluster. (B) UMAPs highlighting the highly localized expression of various cell-type specific marker genes, as expected for robust capture of cell identities in scRNA-seq profiles.

bioRxiv preprint doi: https://doi.org/10.1101/2023.11.28.569014; this version posted June 20, 2024. The copyright holder for this preprint (which was not certified by peer review) is the author/funder, who has granted bioRxiv a license to display the preprint in perpetuity. It is made available under aCC-BY 4.0 International license.

A

	G_1 enriched	S enriched	G_2M enriched
Atrichoblast	74	74	74
Atrichoblast / Epidermis	15	15	15
Atrichoblast Mature	20	20	20
Cortex	12	12	12
Endocortex	14	14	14
Endodermis	3	3	3
Epidermis / Trichoblast / LRC / Columella	89	89	89
G_2M	128	37	554
G_2M / Epidermis	8	8	8
LRC / Columella	186	186	186
Pericycle / Phloem	28	28	28
QC / Initials / Endodermis	18	18	18
QC / Initials / Stele	115	115	115
Stele	23	23	23
Trichoblast	45	45	45
Xylem / Young Stele	36	36	36
Young LRC / Columella	169	169	169
Young Epidermis	112	112	112

B

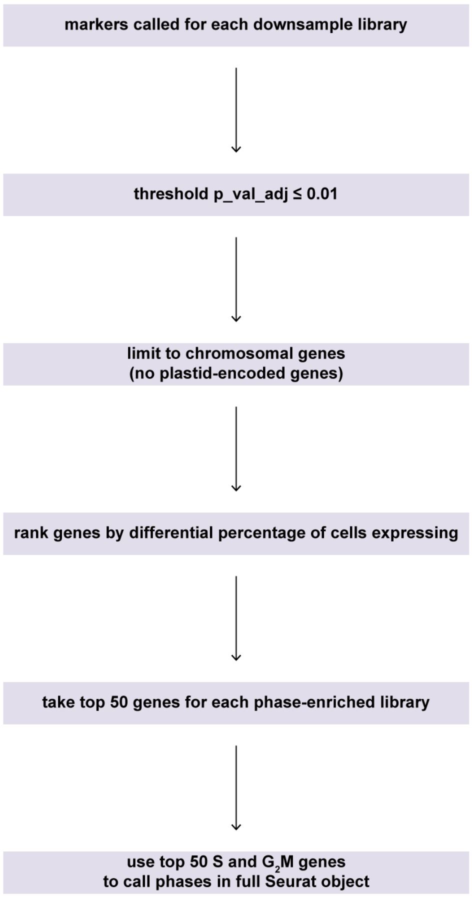

C

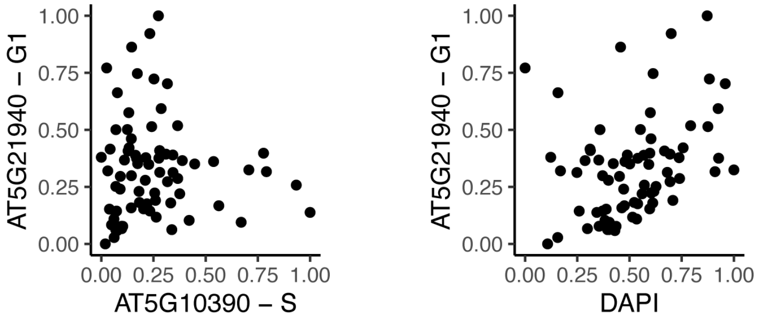

Figure S3. Data analysis methods identify cell phase markers with *in situ* validation of a new G1 marker; Related to Figure 1. (A) Cell counts for down-sampled phase-enriched libraries, ensuring a cell type contributed an equal number of cells to phase enrichment analysis and each cell type contributed to phase enrichment analysis. (B) Differential expression analysis pipeline to identify phase markers. (C) Anti-correlation between the G1 HCR probe and S-phase probe (left) and control plot showing G1 probe and DAPI signal with no anti-correlation (right).

bioRxiv preprint doi: https://doi.org/10.1101/2023.11.28.569014; this version posted June 20, 2024. The copyright holder for this preprint (which was not certified by peer review) is the author/funder, who has granted bioRxiv a license to display the preprint in perpetuity. It is made available under aCC-BY 4.0 International license.

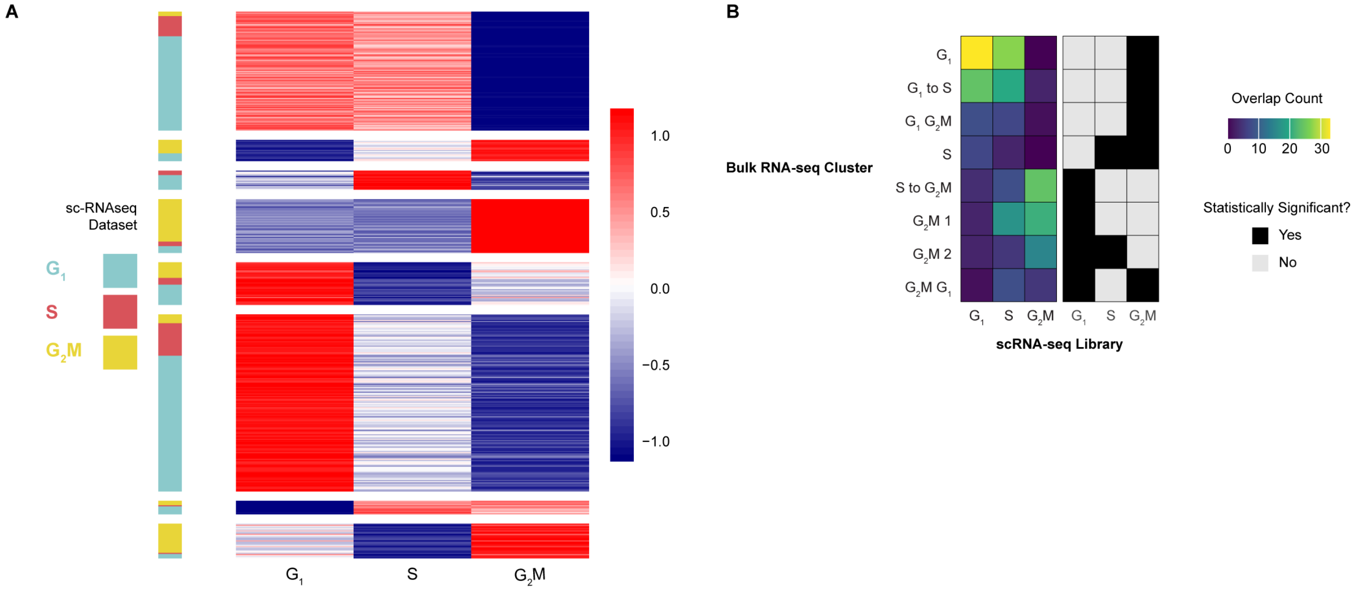

<u>Figure S4:</u> Bulk RNA-seq profiles of the cell cycle confirm phase-enriched scRNA-seq; Related to Figure 1.

(A) Gene expression heatmap (red and blue) in which each row is a gene and each column represents the average expression profile across bulk RNA-seq profiles. Cells were sorted by phase using FACS to determine cellular ploidy level. The color bar to the left indicates which phase-enriched scRNA-seq library a given gene was upregulated in. Genes are grouped into 8 k-means clusters. High overlaps are shown for G1 and G2/M, while S-phase is not well defined in the ploidy sorting (B) Heatmaps showing the number of overlapping genes (left) and the statistical significance of the overlap (right) between differentially expressed genes from phase-enriched scRNA-seq (columns) and gene expression clusters of ploidy-sorted cells determined by k-means clustering (rows). Yes=statistically significant overlap at $p<0.05$. See also Table S4.

bioRxiv preprint doi: https://doi.org/10.1101/2023.11.28.569014; this version posted June 20, 2024. The copyright holder for this preprint (which was not certified by peer review) is the author/funder, who has granted bioRxiv a license to display the preprint in perpetuity. It is made available under aCC-BY 4.0 International license.

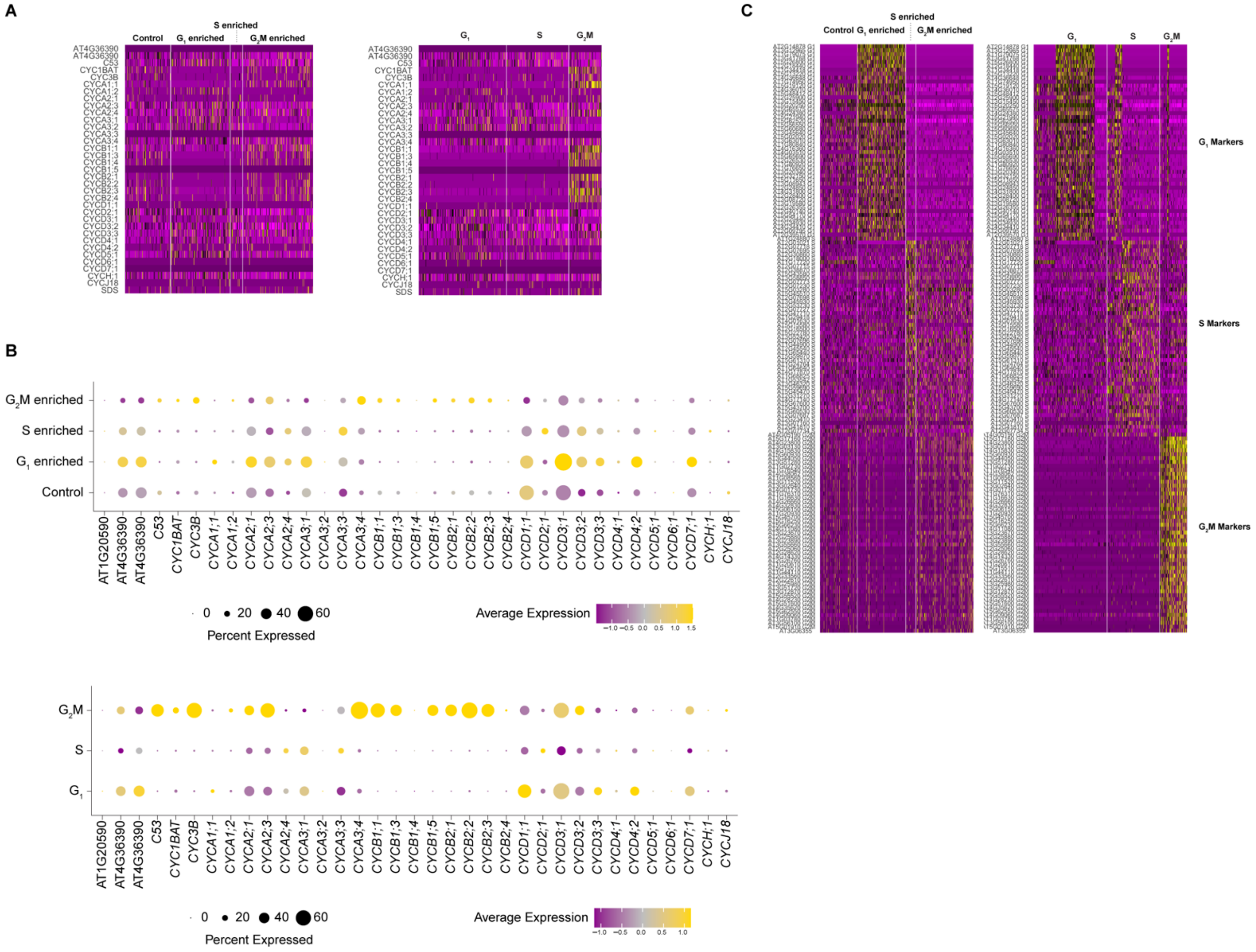

<u>Figure S5:</u> Enrichment analysis for phase markers shows agreement with known cell cycle markers but identifies more robust markers; Related to Figure 1. (A) Heatmaps comparing expression of classical cell-cycle markers (rows) in cells (columns) grouped by the phase enrichment library from which they came (left) vs. cells assigned to phase based on marker analysis (right). At left, some enrichment of markers is visible but phase enriched libraries still contain cells in the non-target phase. At right, enrichment of known markers is more prominent when cells are grouped by our analysis pipeline, which is independent of the expression of the classical cell cycle markers. (B) A summary analysis of the heatmap data in A. Dotplots show the expression of cyclins in phase-enriched libraries (top) vs phases assigned with our top marker genes (bottom). Cyclins are expressed in the appropriate datasets despite their sparseness (top). Cyclin expression behaves well based on phase assignments performed with our marker genes (bottom). (C) Following the same comparison as in A with the top 50 markers assigned by our pipeline. At left, the markers are shown based on their enrichments in the different phase libraries. These agree with classical markers but the analysis shows the new markers have higher expression and are more frequently detected in single-cell profiles. At right, the analysis show cells grouped into now phases using the top 50 markers. Note that many G1-phase markers also express in early S phase, but S-phase has distinct markers to separate G1 and early S.

bioRxiv preprint doi: https://doi.org/10.1101/2023.11.28.569014; this version posted June 20, 2024. The copyright holder for this preprint (which was not certified by peer review) is the author/funder, who has granted bioRxiv a license to display the preprint in perpetuity. It is made available under aCC-BY 4.0 International license.

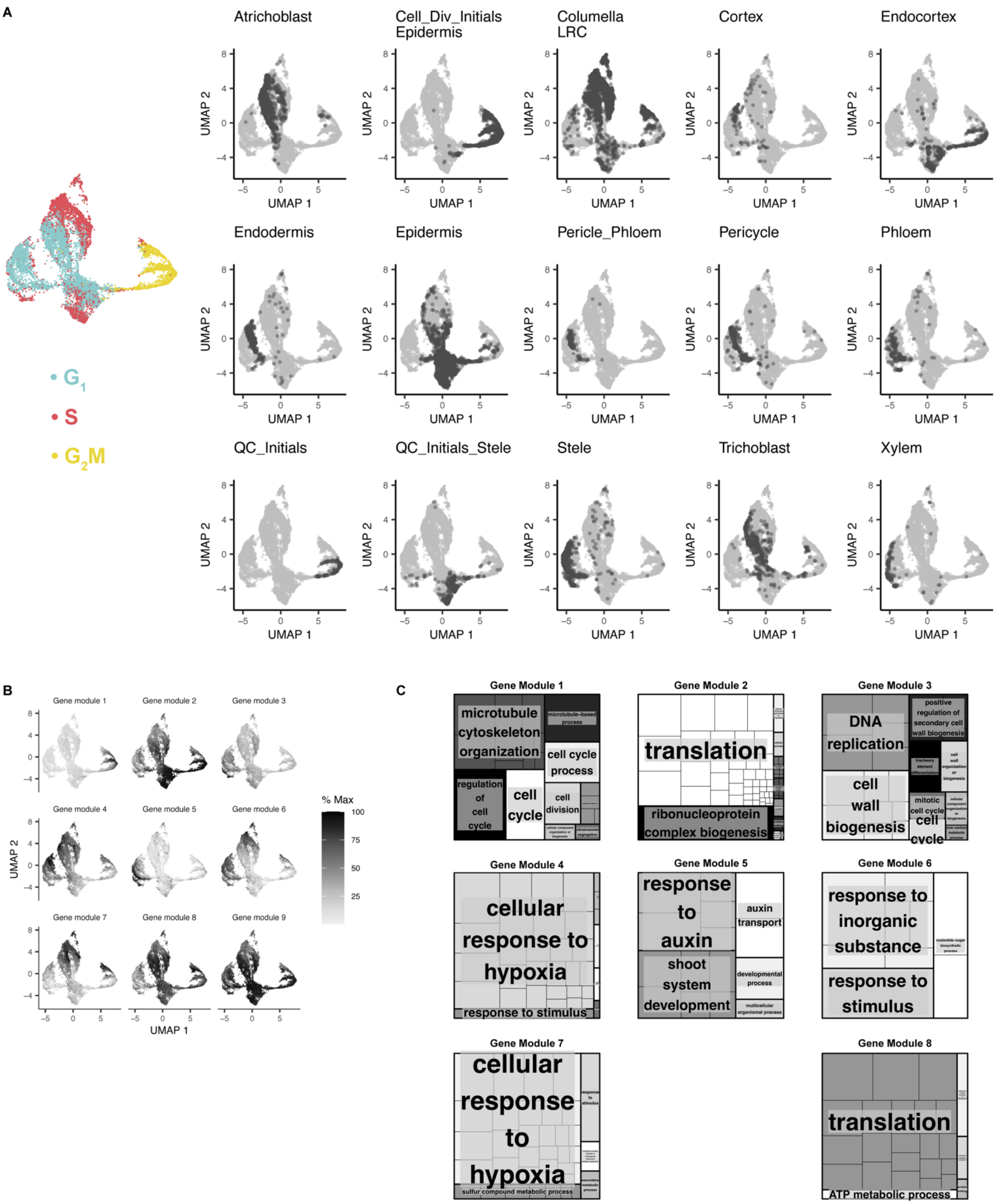

<u>Figure S6</u>. Cells of the same identity group together even when clustered by only cell cycle markers; Related to Figure 2. (A) UMAP outputs of pseudotime analysis clustered using the top 50 cell-cycle markers with an independent analysis of cell identity mapped onto the UMAP trajectories. In each panel, a different cell type is highlighted in black. At left, the cell cycle classifications are shown. (B) Analysis of gene modules that are preferentially expressed in dominant intervals along the pseudotime ordering, as determined by Monocle3

bioRxiv preprint doi: https://doi.org/10.1101/2023.11.28.569014; this version posted June 20, 2024. The copyright holder for this preprint (which was not certified by peer review) is the author/funder, who has granted bioRxiv a license to display the preprint in perpetuity. It is made available under aCC-BY 4.0 International license.

(see Methods). Grayscale shows the aggregate gene expression of each gene module. (C) GO-terms associated with the corresponding gene module shown in B. No significant GO terms were found for gene module 8.

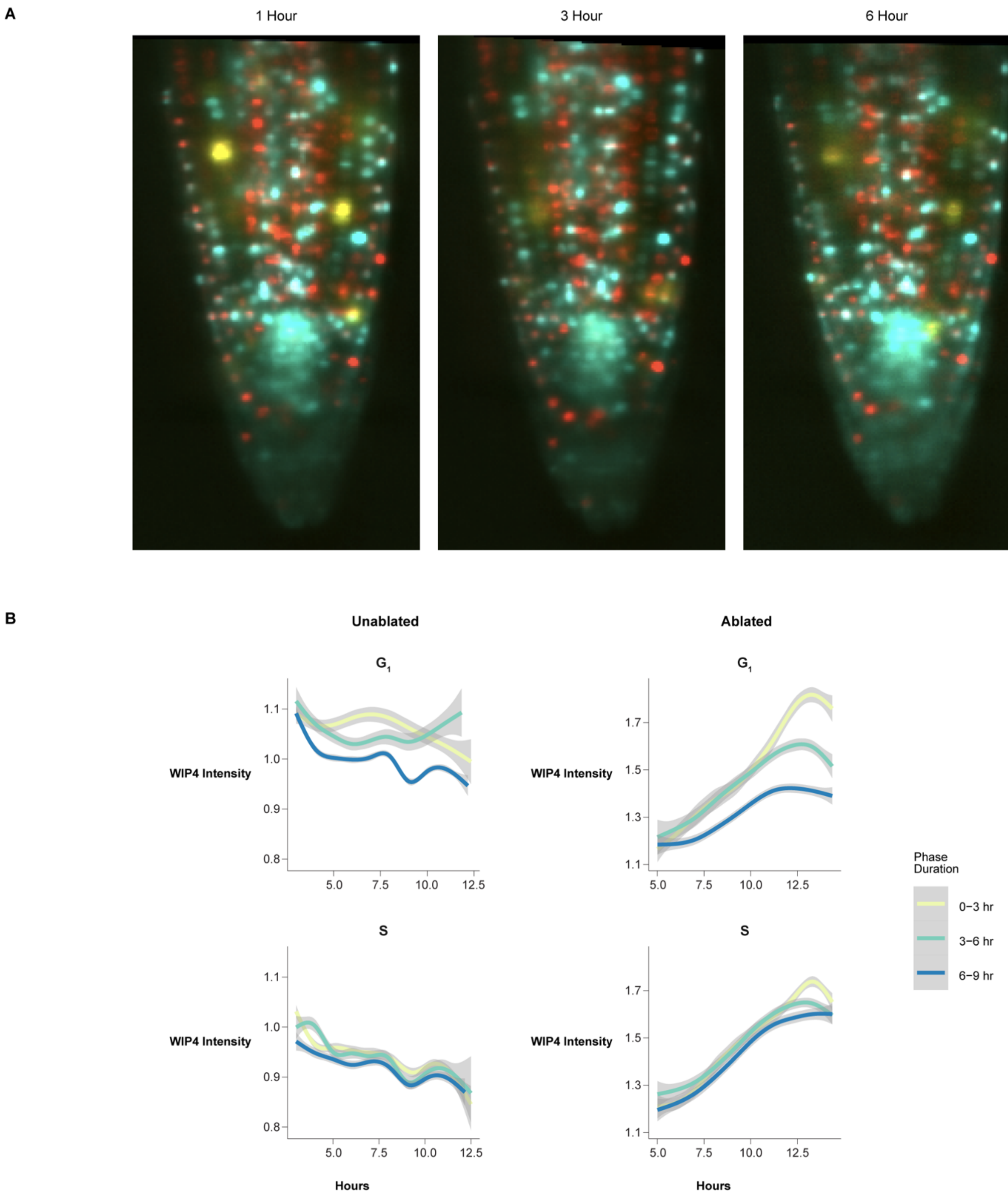

<u>Figure S7:</u> The appearance of newly reprogrammed cell identity correlates with rapid G1 phases; Related to Figure 3. (A) Representative images of a control root expressing PlaCCI and WIP4::GFP at 1, 3, and 6 hour time points during a time-lapse acquisition. (B) Quantification of the WIP4 signal intensity in CFP+ and mCherry+ cells over the duration of time-lapse movies. The figure represents the complete analysis of data shown in Figure 3E.

bioRxiv preprint doi: https://doi.org/10.1101/2023.11.28.569014; this version posted June 20, 2024. The copyright holder for this preprint (which was not certified by peer review) is the author/funder, who has granted bioRxiv a license to display the preprint in perpetuity. It is made available under aCC-BY 4.0 International license.

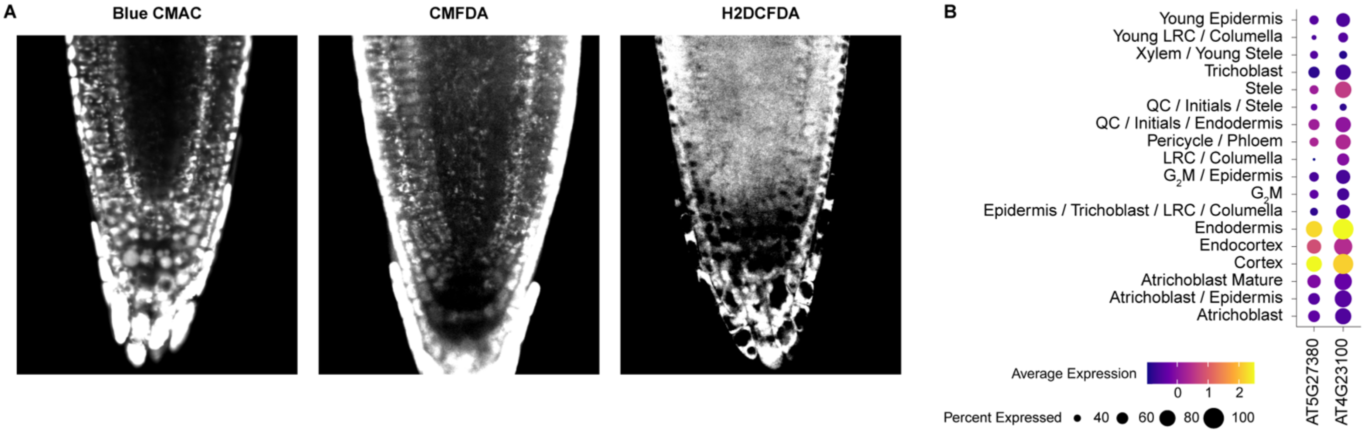

<u>Figure S8.</u> ROS and GSH dyes show different tissue localization patterns; Related to Figure 4. (A) Representative confocal microscopy images of seedlings stained for GSH (blue CMAC, CMFDA) or ROS (H2DCFDA) under control conditions. Note that the two GSH dyes agree and show prominent ground tissue staining. Note that CMFDA and H2DCFDA, with similar chemical structure but different target molecules, show different staining patterns. (B) Expression of GSH1 and GSH2 represented as dot plot derived from scRNA-seq profiles in different root cell types. Note the prominent expression in endodermis and cortex, in agreement with the GSH dyes.

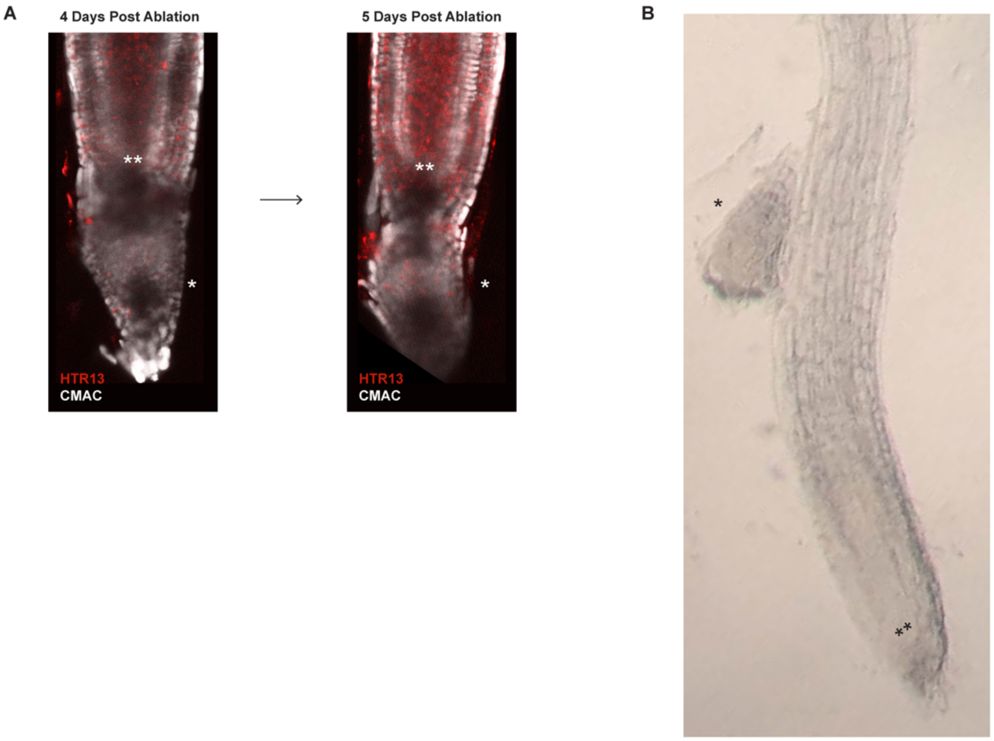

<u>Figure S9</u>. Transverse ablation leads to the reformation of a new root tip similar to the root tip excision procedure; Related to Figure 4. (A) Representative confocal images of seedlings (grown on standard ½ MS and then mounted in an imaging cuvette) undergoing regeneration. Between days 4 and 5 post ablation it becomes apparent that new columella above the ablation is established proximal (shootward) to the original

bioRxiv preprint doi: https://doi.org/10.1101/2023.11.28.569014; this version posted June 20, 2024. The copyright holder for this preprint (which was not certified by peer review) is the author/funder, who has granted bioRxiv a license to display the preprint in perpetuity. It is made available under aCC-BY 4.0 International license.

QC (*), which is below the ablation. The tapered root cap, which includes the columella, is apparent distal to the new QC (**), both of which are above the ablation site. (B) At a later time point, the original root tip (*) is sloughed off as growth continues from the new QC/stem cell niche (**) in the same seedling shown in the lower panel of A.

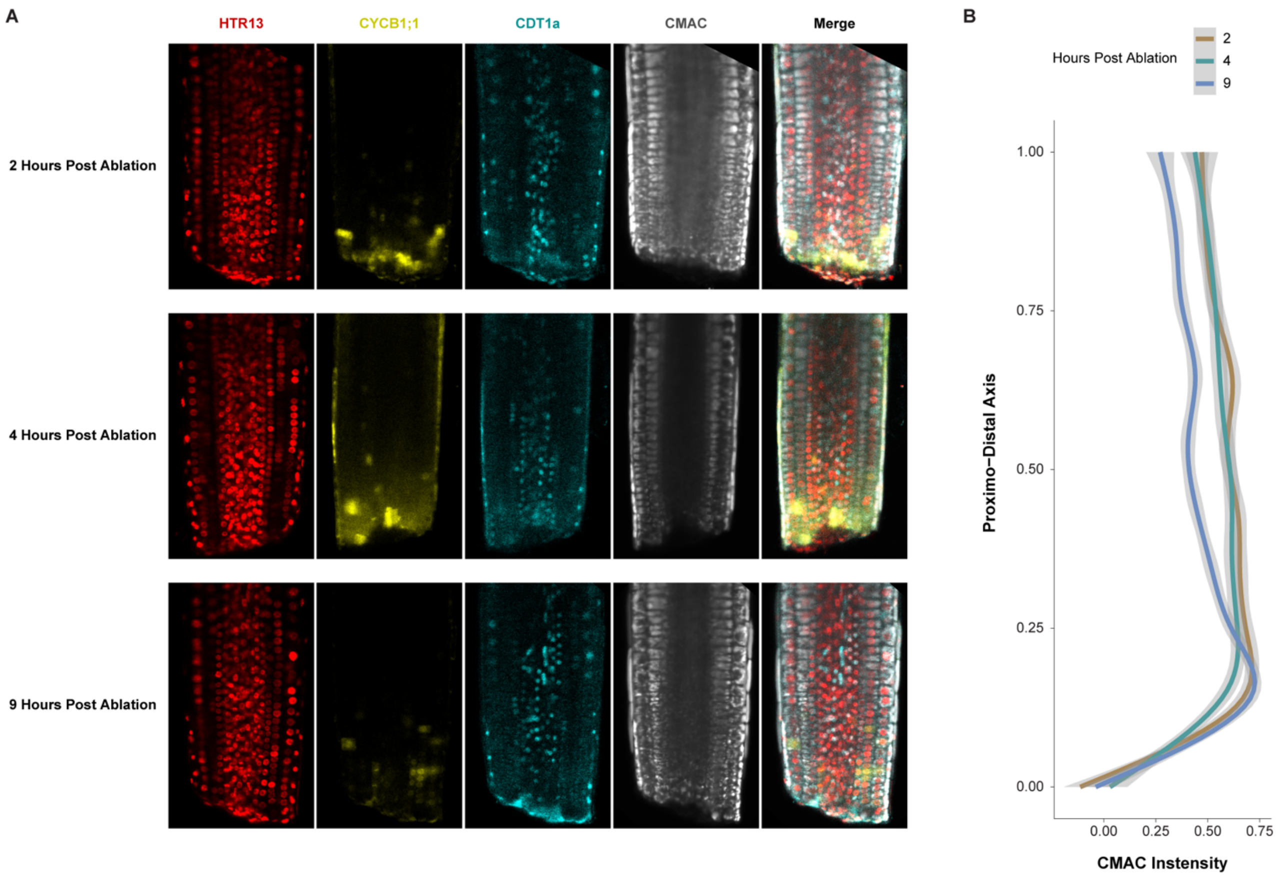

<u>Figure S10.</u> GSH dye CMAC is brightest in the same region where cells undergo rapid division and shortened G1 during regeneration; related to Figure 4. (A) Representative confocal images of PlaCCI roots stained with blue CMAC. Images were taken 2, 4, and 9 HPC. (B) Quantification of nuclear CMAC staining intensity along the proximal-distal axis at different time points after ablation. The y-intercept represents the ablation site and the range of the y-axis represents the visible length of root imaged in the frame as shown in A. Note the peak of CMAC intensity right above the cut site between 0.00 and 0.25 on the longitudinal axis of the root (y-axis), which is highest at 2-4 hrs post cut and begins to dissipate above point 0.25 at 9 hrs.
