# Supplementary material for: Nuclear GSH import precedes coordinated cell cycle changes during regeneration
Source: bioRxiv. 2023 Dec 13:2023.11.28.569014. Preprint. [Version 2] doi: 10.1101/2023.11.28.569014 (PMC10760015; doi:10.1101/2023.11.28.569014)
Supplement: Supplementary file 1 [file media-3.pdf]

Supplemental Figures

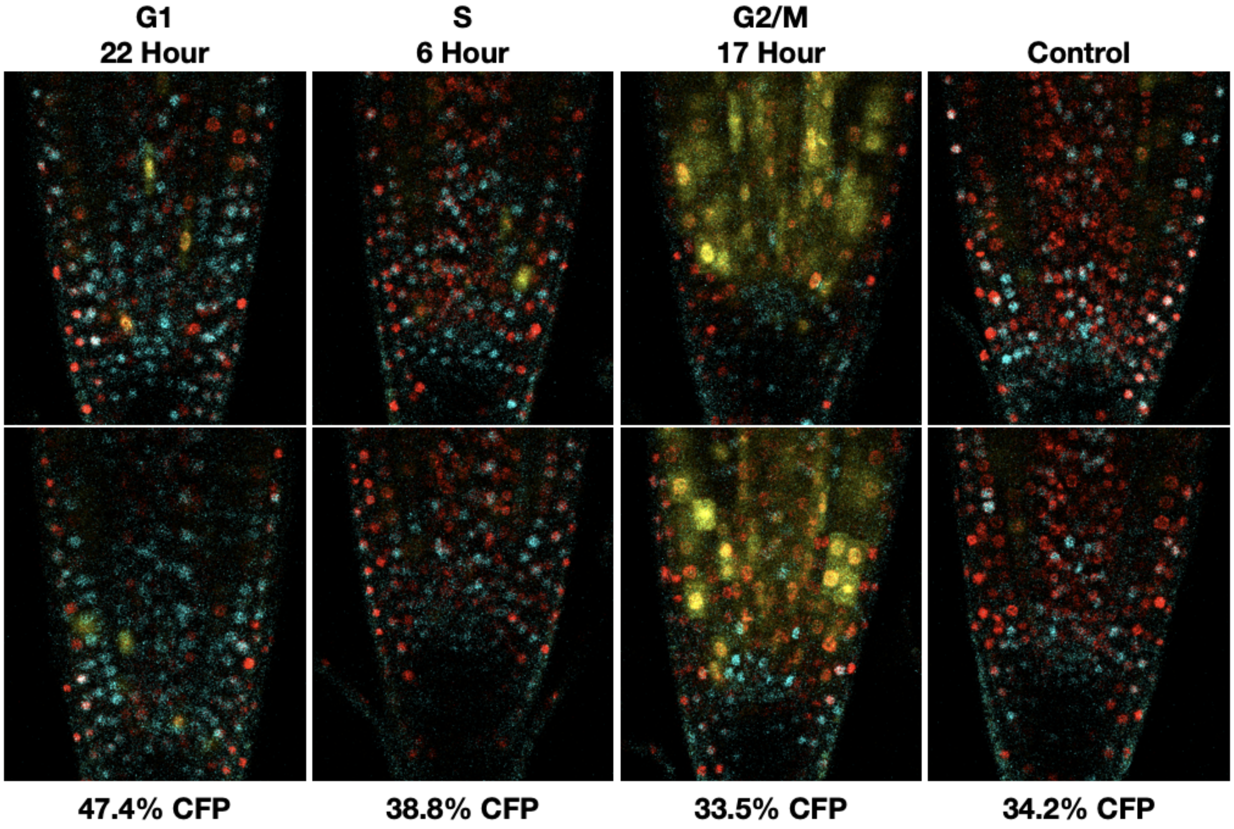

Supplemental Figure 1: Seedlings were grown on ½ MS plates with 2mM HU for the indicated periods of time. The fraction of nuclei that were CFP+ is shown below for Z stacks segmented in 3D using Imaris.

A

	G1_enriched	S_enriched	G2M_enriched
Atrichoblast	74	74	74
Atrichoblast_epidermis	15	15	15
Atrichoblast_mature	20	20	20
Cortex	12	12	12
Endocortex	14	14	14
Endodermis	3	3	3
Epidermis_Trichoblast_LRC_Columella	89	89	89
G2M	128	37	554
G2M_Epidermis	8	8	8
LRC_Columella	186	186	186
Pericycle_Phloem	28	28	28
QC_Initials_Endodermis	18	18	18
QC_Initials_Stele	115	115	115
Stele	23	23	23
Trichoblast	45	45	45
Xylem_Young_Stele	36	36	36
Young LRC_Columella	169	169	169
Young_Epidermis	112	112	112

B

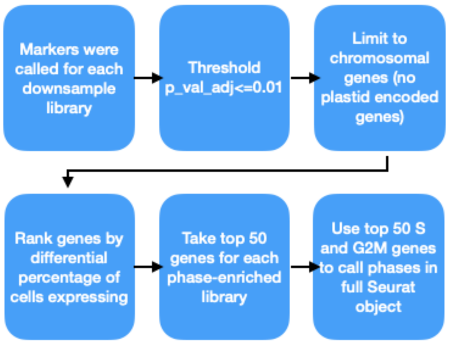

<u>Supplemental Figure 2:</u> A. Cell counts for downsampled phase-enriched libraries. B. Differential expression testing pipeline for phase markers.

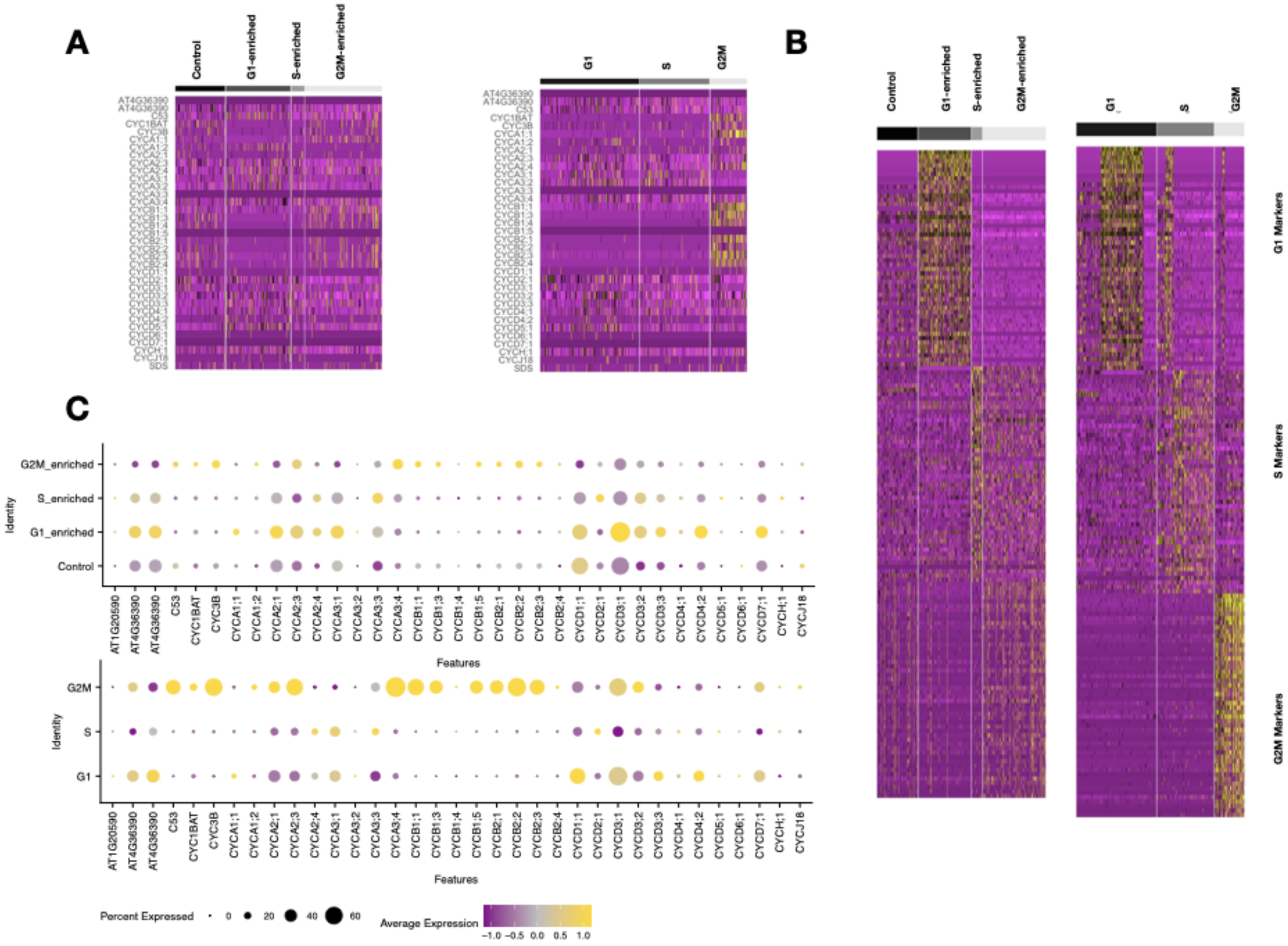

Supplemental Figure 3: A. Heatmaps showing the expression of cyclins across all cells in phase-enriched libraries (left) and phases assigned with our top marker genes (right). Cyclin expression is sparse. B. Heatmaps showing the expression of our phase markers in phase-enriched libraries (left) and phases assigned with our top marker genes (right). Genes are more broadly expressed. C. Dotplots showing the expression of cyclins in phase-enriched libraries (top) and phases assigned with our top marker genes (bottom). Cyclins are expressed in the appropriate datasets despite their sparseness (top). Cyclin expression behaves well based on phase assignments performed with our marker genes (bottom).

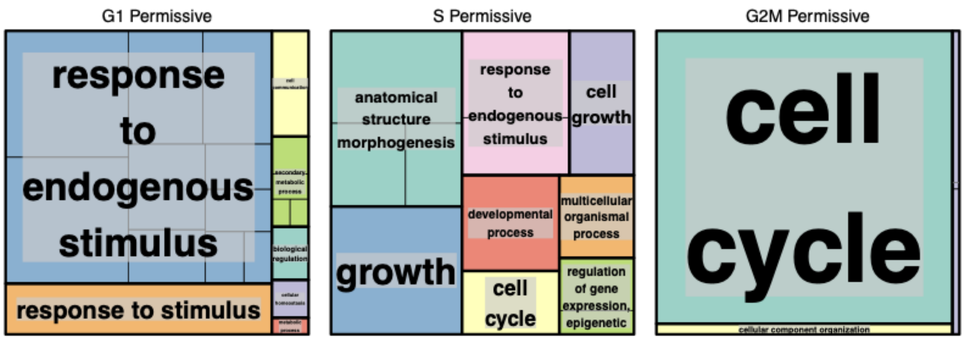

Supplemental Figure 4: Enriched GO terms for the top 200 marker genes for each phase enriched scRNA-seq library.

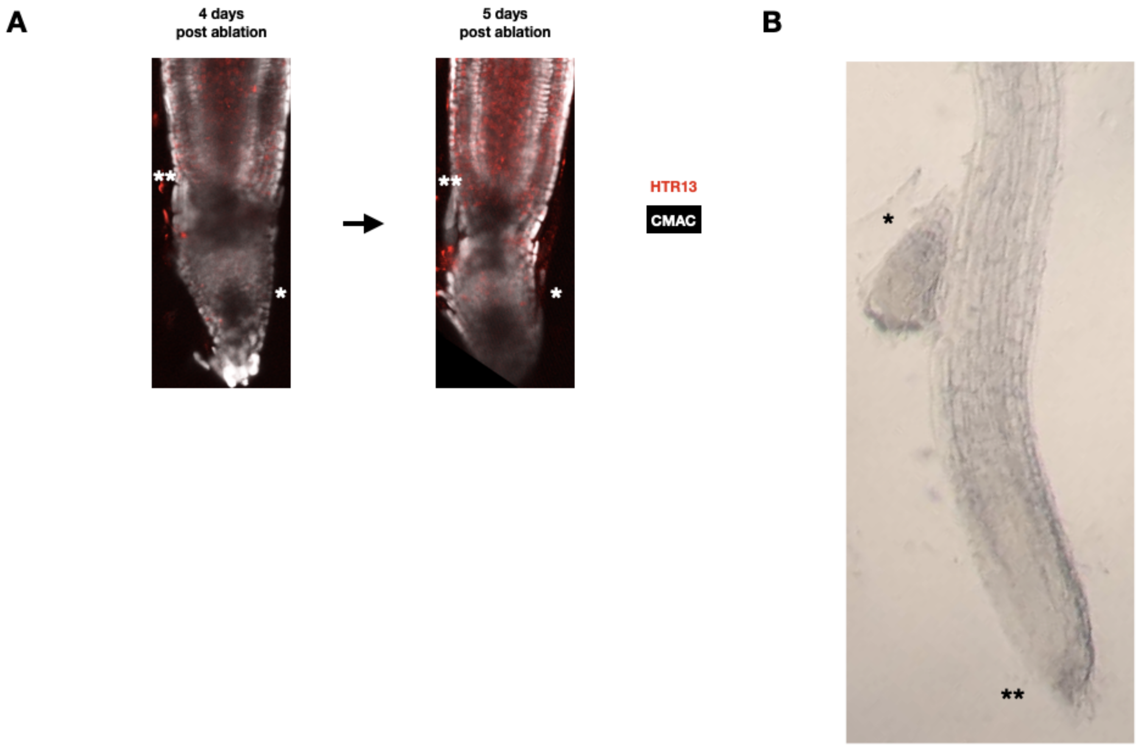

<u>Supplemental Figure 5:</u> A. Representative confocal images of seedlings grown on normal ½ MS and then mounted in an imaging cuvette undergoing regeneration. Between days 4 and 5 post ablation it becomes apparent that new columella is established proximal to the original QC (*) and distal to the new QC (**). B. The original root tip (*) is lost as growth continues from the new QC (**) in the same seedling shown in the lower panel of A.

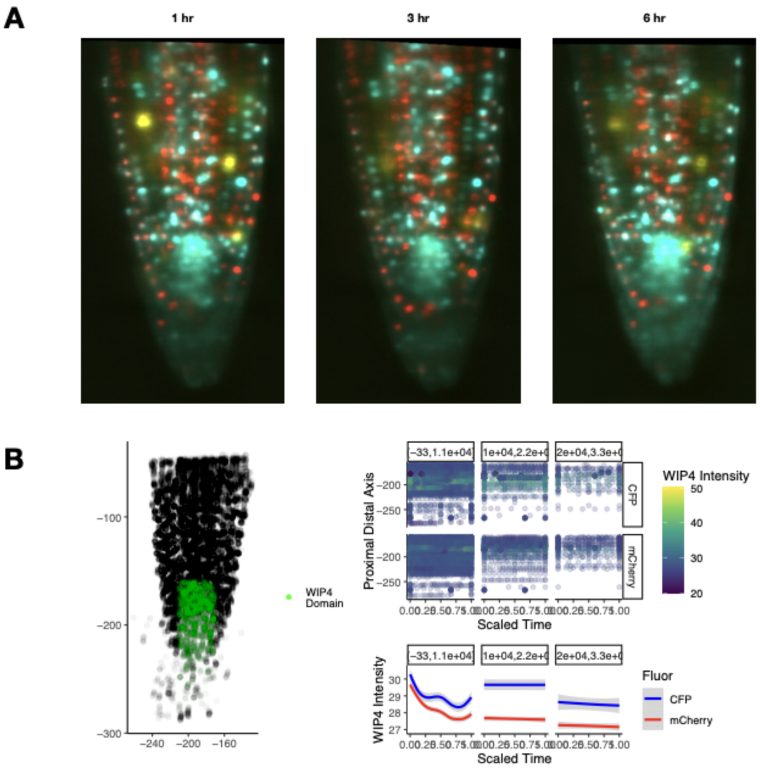

Supplemental Figure 6: A. Representative images of a seedling expressing PlaCCI and WIP4::GFP at 1, 3, and 6 hour time points during a time lapse acquisition. B. Quantification of the WIP4 signal intensity in CFP+ and mCherry+ cells over the duration of a time lapse movie.

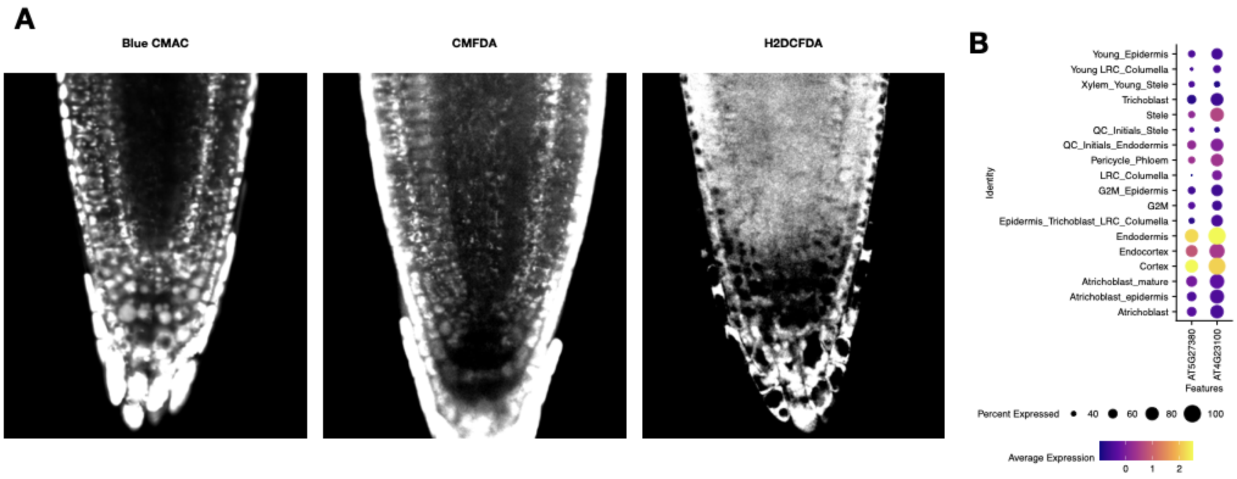

Supplemental Figure 7: A. Representative confocal microscopy images of seedlings stained for GSH (blue CMAC, CMFDA) or ROS (H2DCFDA) under control conditions. B. Expression of GSH1 and GSH2 in root cell types.

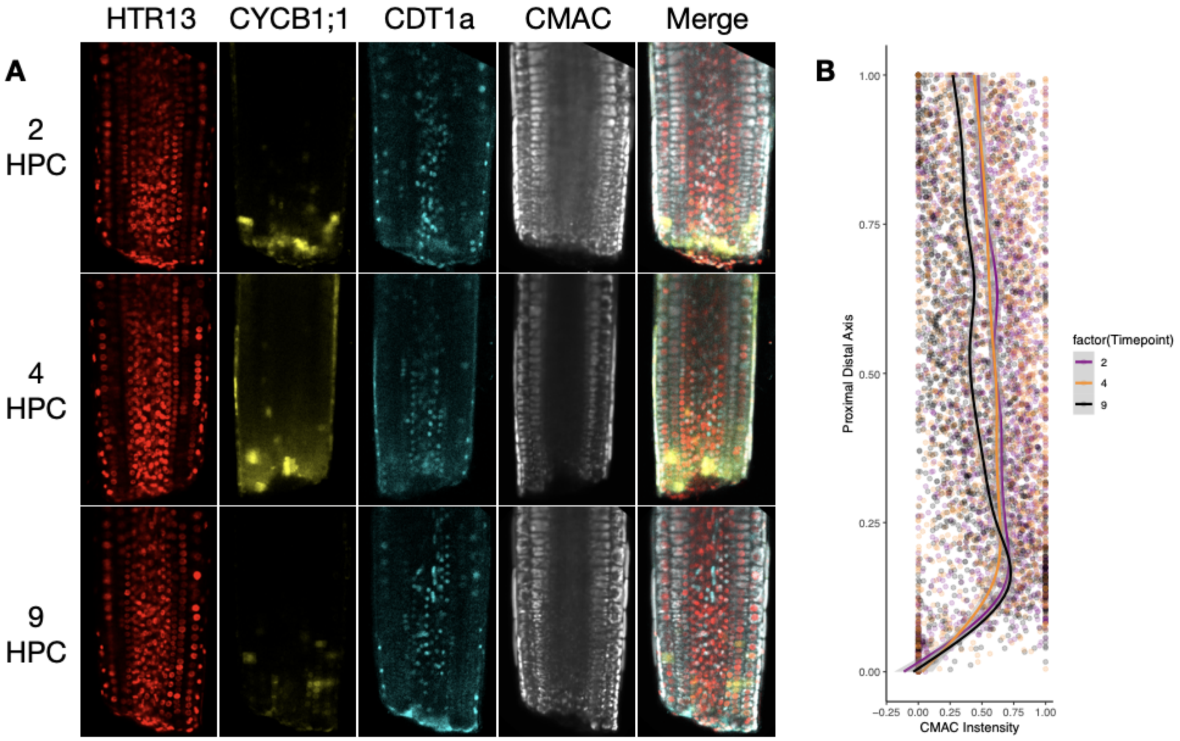

<u>Supplemental Figure 8</u> A. Representative confocal images of roots expressing PlaCCI and stained with blue CMAC. Images were taken 2, 4, and 9 HPC. B. Quantification of nuclear CMAC staining intensity along the proximal-distal axis.
